# Classical monocyte-derived macrophages as therapeutic targets of umbilical cord mesenchymal stem cells: comparison of intratracheal and intravenous administration in a mouse model of pulmonary fibrosis

**DOI:** 10.1186/s12931-023-02357-x

**Published:** 2023-03-05

**Authors:** Sun Mi Choi, Yosep Mo, Ji-Young Bang, Young Gyun Ko, Yoon Hae Ahn, Hye Young Kim, Jaemoon Koh, Jae-Joon Yim, Hye-Ryun Kang

**Affiliations:** 1 Division of Pulmonary and Critical Care Medicine, Department of Internal Medicine, Seoul National University Hospital, Seoul, Republic of Korea; 2 Department of Translational Medicine, Seoul National University College of Medicine, Seoul, Republic of Korea; 3 Laboratory of Mucosal Immunology, Department of Biomedical Sciences, Seoul National University College of Medicine, Seoul, Republic of Korea; 4 Department of Critical Care Medicine, Seoul National University Hospital, Seoul, Republic of Korea; 5 Department of Pathology, Seoul National University College of Medicine, Seoul, Republic of Korea; 6 Department of Internal Medicine, Seoul National University College of Medicine, Seoul, Republic of Korea; 7 Institute of Allergy and Clinical Immunology, Seoul National University Medical Research Center, Seoul, Republic of Korea

**Keywords:** Pulmonary fibrosis, Monocyte, Macrophage, Mesenchymal stem cell, Bleomycin

## Abstract

**Background:**

Idiopathic pulmonary fibrosis (IPF) is a progressive fibrotic lung disease that has no cure. Although mesenchymal stem cells (MSCs) have been reported to ameliorate lung inflammation and fibrosis in mouse models, their mechanisms of action remain unknown. Therefore, we aimed to determine the changes in various immune cells, especially macrophages and monocytes, involved in the effects of MSC treatment on pulmonary fibrosis.

**Methods:**

We collected and analyzed explanted lung tissues and blood from patients with IPF who underwent lung transplantation. After establishing a pulmonary fibrosis model via the intratracheal administration of bleomycin (BLM) to 8-week-old mice, MSCs derived from human umbilical cords were administered intravenously or intratracheally on day 10 and the lungs were immunologically analyzed on days 14 and 21. Flow cytometry was performed to analyze the immune cell characteristics, and gene expression levels were examined using quantitative reverse transcription-polymerase chain reaction.

**Results:**

In the histological analysis of explanted human lung tissues, the terminally fibrotic areas contained a larger number of macrophages and monocytes than the early fibrotic areas of the lungs. When human monocyte-derived macrophages (MoMs) were stimulated with interleukin-13 in vitro, the expression of type 2 macrophage (M2) markers was more prominent in MoMs from the classical monocyte subset than in those from intermediate or non-classical monocyte subsets, and MSCs suppressed M2 marker expression independent of MoM subsets. In the mouse model, the increased number of inflammatory cells in the bronchoalveolar lavage fluid and the degree of lung fibrosis observed in BLM-treated mice were significantly reduced by MSC treatment, which tended to be more prominent with intravenous administration than intratracheal administration. Both M1 and M2 MoMs were upregulated in BLM-treated mice. The M2c subset of M2 MoMs was significantly reduced by MSC treatment. Among M2 MoMs, M2 MoMs derived from Ly6C^+^ monocytes were most effectively regulated by the intravenous administration, not intratracheal administration, of MSCs.

**Conclusions:**

Inflammatory classical monocytes may play a role in lung fibrosis in human IPF and BLM-induced pulmonary fibrosis. Intravenous rather than intratracheal administration of MSCs may ameliorate pulmonary fibrosis by inhibiting monocyte differentiation into M2 macrophages.

**Supplementary Information:**

The online version contains supplementary material available at 10.1186/s12931-023-02357-x.

## Background

Pulmonary fibrosis is a sequela of various pathological conditions that can induce fibrotic parenchymal scarring. Idiopathic pulmonary fibrosis (IPF) is characterized by chronic progressive fibrosis and irreversible aberrant tissue remodeling without any known cause, leading to eventual respiratory failure. The standard treatment for IPF includes anti-fibrotics that slow the rate of disease progression; however, there is no treatment modality to cure or reverse fibrosis [1, 2].

Although the exact pathogenesis of IPF is not clearly known, several paradigms of IPF pathophysiology have been proposed, including recurrent epithelial injuries that incite aberrant interactions between alveolar epithelial cells and fibroblasts [3, 4], cellular senescence [5–7], short telomeres [8], inflammation, and immunological mechanisms [9–11]. Several genetic studies have reported the importance of the immune response in IPF and demonstrated its association with immune-related genes, such as Toll-like receptor 3, Toll-interacting protein, and interleukin (IL)-1 receptor [12–15]. In recent years, the role of immune cells in IPF has been extensively studied. There is growing interest in monocytes, as recent studies have reported that a high number of circulating monocytes in the blood is significantly related to disease progression and death in patients with IPF [16–18].

Macrophages are the most abundant immune cells in healthy lungs. Lung macrophages are potent sources of pro-fibrotic cytokines, chemokines, and proteases that contribute to the pathogenesis of pulmonary fibrosis [19]. The major lung macrophage population consists of alveolar macrophages (AMs), which populate the alveolar and airway lumen, and interstitial macrophages present in the lung interstitium. AMs can be further divided according to their origin as tissue-resident AMs (TR-AMs), which originate from the yolk sac or fetal liver and reside in the lungs to maintain homeostasis, and inflammatory monocyte-derived macrophages (MoMs), newly derived from circulating monocytes in the blood by certain environmental cues. Macrophage polarization refers to the process by which activated macrophages produce functionally distinct phenotypes, type 1 (M1) and type 2 (M2) macrophages, in response to signals received from the microenvironment. M1 represents classically activated macrophages that secrete inflammatory cytokines and remove pathogens, while M2 represents alternatively activated macrophages that secrete anti-inflammatory cytokines to heal wounds, terminate inflammatory responses, and contribute to tissue remodeling and fibrosis. Changes in the phenotypic spectrum of M1 and M2 and M2 infiltration have been suggested to lead to the development and progression of pulmonary fibrosis [20]. Furthermore, depletion of macrophages by clodronate liposomes has been reported to attenuate bleomycin (BLM)-induced pulmonary fibrosis, suggesting that macrophages play a critical role in the development of pulmonary fibrosis [21, 22]. In addition, adoptive transfer of bone marrow-derived M2 into clodronate liposome-treated mice induced severe pulmonary fibrosis following BLM administration, suggesting that M2 macrophages play a vital role in pulmonary fibrosis [22]. Other studies have also shown that inhibition of M2 macrophages improves pulmonary fibrosis, indicating their potential as therapeutic targets for IPF [23, 24].

Mesenchymal stem cells (MSCs) are stromal cells that exist in the cartilage matrix, bone, fat, bone marrow, and tissues derived from the mesoderm. MSCs commonly exhibit stemness, self-renewal capacity, and plasticity, allowing them to proliferate and differentiate into several types of cells that make up organs under specific circumstances. MSCs have different characteristics, depending on their origin. Umbilical cord blood- and placenta-derived MSCs exhibit good plasticity and low immunogenicity.

MSCs have unique properties that facilitate their use as potential therapeutic agents for various diseases. MSCs play vital roles in tissue repair and regeneration by generating differentiated cells [25, 26]. They also regulate the immune system by modulating the activity of various immune cells, affecting the differentiation of macrophages, and secreting various cytokines [27, 28]. In addition, MSCs exert paracrine, anti-inflammatory, and anti-fibrotic effects by secreting or suppressing the secretion of several cytokines and growth factors [28].

In a BLM-induced pulmonary fibrosis model, MSC treatment was reported to decrease the expression levels of IL-10, interferon (IFN)-γ, and tumor necrosis factor (TNF)-α as well as the concentration of transforming growth factor (TGF)-β1 in the lungs, resulting in decreased collagen expression [29]. Other studies have also reported similar anti-fibrotic effects of MSC treatment in murine pulmonary fibrosis models [30–34]. MSC treatment has been reported to decrease inflammation and collagen deposition in the lung tissues of fibrosis murine models via several mechanisms, such as replacement of type II pneumocytes differentiated from MSCs [31], regulation of immune cells by extracellular vesicles (EVs) [34], and down-regulation of nitric oxide metabolites and proinflammatory and angiogenic cytokines [32]. These results suggest that MSCs can potentially be used as promising therapeutic agents [35–38]. Despite the significant interest in MSCs, studies focusing on the effects of MSCs on macrophages and monocytes, especially in association with pulmonary fibrosis, are minimal compared to other organs [30–34]. Numerous animal studies and clinical trials have demonstrated the regenerative, anti-fibrotic, and immune regulatory effects of MSC treatment in various liver diseases, including liver cirrhosis, acute liver failure, and autoimmune hepatitis [39]; various neurological diseases, including Parkinson’s disease, Alzheimer’s disease, and amyotrophic lateral sclerosis [40] and various kidney diseases, including renovascular disease, sepsis-associated acute kidney injury, and diabetic kidney disease [41]. Adipose tissue- and bone marrow-derived MSCs have been reported to exert protective effects against other lung diseases, such as acute respiratory distress syndrome and chronic obstructive pulmonary disease [42].

In this study, we aimed to understand the roles of monocytes and macrophages in the development of pulmonary fibrosis and investigate the effects of MSCs on immune cells, especially monocytes and macrophages, to suppress lung fibrosis using a BLM-induced lung fibrosis mouse model.

## Methods

### Lung tissue and blood sampling from patients with IPF

We collected explanted lung tissues from five patients with IPF who underwent lung transplantation and blood from one patient with advanced IPF. For histological comparison, we collected two different areas of the lungs from each patient in accordance with chest computed tomography imaging findings as follows: one piece with little fibrotic change that maintained alveolar space from the upper lobes for the early fibrotic area, and the other piece with severe fibrotic and honeycombing changes from the lower lobes for the terminally fibrotic area. A lung pathologist confirmed the hematoxylin and eosin (H&E)-stained slides to ensure the relevance of sampling. Structural integrity was also confirmed using H&E- and Masson's trichrome (MT)-stained slides. The degrees of inflammatory cell infiltration and fibrosis were evaluated by a pathologist. This study was approved by the Institutional Review Board of the Seoul National University Hospital (IRB No. H02204-146-1319) and conducted in accordance with the Declaration of Helsinki.

### Mouse model of pulmonary fibrosis

We established a mouse model of pulmonary fibrosis using 8-week-old female C57BL/6 mice purchased from Orient Bio (Anyang, South Korea). For intratracheal administration of BLM, the mice were anesthetized with 3% isoflurane and fixed perpendicular to the floor. The tongue of the mouse was pulled back using forceps to block the esophagus and expose the airway. A total of 3 mg/kg BLM (Nippon Kayaku, Tokyo, Japan) was intratracheally administered using a pipette, and the same amount of phosphate-buffered saline (PBS; Biowest, France) was intratracheally administered to the control group. The experiments were approved by the Institutional Animal Care and Use Committee of the Institute of Laboratory Animabl Resources of Seoul National University (SNU-200327-1-3).

### Analysis of inflammation and histology

We performed bronchoalveolar lavage (BAL) in mice to analyze the lung inflammatory response and sacrificed them on days 14 and 21 to analyze the lung tissues. For BAL, the mice were anesthetized, a catheter was intubated into the upper respiratory tract, and 0.9 mL of PBS was administered twice to obtain the BAL fluid (BALF). Cells obtained from BALF were stained with Diff-Quik (Systmex, Kobe, Japan), and the numbers of macrophages, neutrophils, eosinophils, and lymphocytes within the sample were counted. Lung tissue samples were fixed and embedded in paraffin and stained with H&E, MT, terminal deoxynucleotidyl transferase dUTP nick-end labeling (TUNEL), and periodic acid-Schiff (PAS) stains. Inflammation, fibrosis, apoptosis, and mucus production in the lungs were semi-quantified and scored according to previous studies [43–46].

### Flow cytometry

Chopped human and mouse lung tissues were incubated with type IV collagenase (Worthington Biochemical Corporation, Freehold, NJ, USA) at 37 °C for 30 and 90 min, respectively. Then, the separated single cells were filtered through sterile 40 μm strainers and red blood cells (RBCs) were removed using the RBC lysis buffer (Sigma Aldrich, Burlington, MA, USA).

Filtered lung single-cell suspensions were treated with FcγR-blocking monoclonal antibodies (BD Biosciences, Franklin Lakes, NJ, USA). Cell surface markers, intracellular cytokines, and transcription factors were stained accordingly, and each immune cell was gated according to the supplementary method (Additional file 1: Figs. S1–S3; Table S1).

### Macrophage differentiation from monocytes isolated from human peripheral blood mononuclear cells (PBMCs)

Human PBMCs extracted from patients with IPF were separated from blood diluted with PBS via 1800 RPM centrifugation using Ficoll-Paque PLUS (GE Healthcare, Uppsala, Sweden). For the isolation of monocyte subtypes, isolated PBMCs were sorted using a BD AriaIII (BD Biosciences). Isolated monocytes were differentiated into macrophages by treatment with 50 ng/mL recombinant human macrophage colony-stimulating factor (M-CSF; BioLegend, San Diego, CA, USA). MSCs were treated with M-CSF on day 0. Five days after M-CSF treatment, differentiated macrophages were treated with 20 ng/mL IL-13 for one day.

### Ex vivo immune cell culture and macrophage differentiation from murine monocytes

For CD4^+^ T cell isolation, filtered murine splenocyte suspensions were negatively selected using the MojoSort Mouse CD4 Naive T Cell Isolation Kit (BioLegend). To determine the effect of MSCs on modulating the Th17 dominant immune response, CD4^+^ T cells isolated from the mouse spleen were differentiated using the CellXVivo Mouse Th17 Cell Differentiation Kit (rndsystems, Minneapolis, MN, USA), according to the manufacturer's instructions, and then incubated with MSCs in the lower chamber of a 24-well transwell plate.

For murine monocyte isolation, filtered bone marrow single-cell suspensions retrieved from the BLM model were negatively selected using the MojoSort Mouse Monocyte Isolation Kit (BioLegend). Negatively isolated monocytes were stained with PE-conjugated Ly6c antibody to classify them as Ly6c^+^ or Ly6c^–^ monocytes using the MojoSort Mouse anti-PE nanobeads (Biolegend). Isolated monocytes were differentiated into macrophages by treatment with 25 ng/mL M-CSF (BioLegend). MSCs were treated with M-CSF on day 0. Five days after M-CSF treatment, differentiated macrophages were treated with 20 ng/mL IL-13 for one day.

### Ex vivo model to evaluate the regulation effect of Ly6c^+^ MoMs on CD4^+^ T cell plasticity

Monocytes isolated from the bone marrow of the BLM model were differentiated into macrophages by treatment with 25 ng/mL M-CSF (BioLegend) on day 0, and MSCs were administered on day 3. Five days after M-CSF treatment, differentiated Ly6c^+^ MoMs were co-cultured with naive CD4^+^ T cells derived from naïve mouse spleens for two days to evaluate the regulation effect of Ly6c^+^ MoMs on Th2 and Th17 plasticity. Naive CD4^+^ T cells that were not co-cultured were used as the control group.

### In vitro fibrosis model

For the in vitro human and mouse lung fibrosis models, CCL-117 (human lung fibroblasts) and MLg (mouse lung fibroblasts) cells were purchased from the Korea Cell Line Bank (Seoul). Cells were cultured at 37 °C by adding 10% fetal bovine serum (Biowest) to Dulbecco's modified Eagle’s medium (Biowest), and the medium was changed once every three days. Induction of fibrosis was evaluated by transferring a culture medium of human- or mouse-derived MoMs to fibroblasts (2 × 10^4^) together with recombinant TGF-β protein at a concentration of 20 ng/mL.

### Reverse transcription-quantitative polymerase chain reaction (RT-qPCR)

Frozen human and mouse lung tissues were homogenized and RNA was isolated using TRIzol (Thermo Fisher Scientific Inc., Waltham, MA, USA) and chloroform. To synthesize complementary DNA (cDNA), the isolated RNA was reverse-transcribed using the SensiFAST cDNA Synthesis Kit (Bioline, London, UK). RT-qPCR was performed using the SensiFAST SYBR No-ROX Kit (Bioline) and the relative expression of the target gene was determined via the ΔΔ*C*_*t*_ method using a housekeeping gene (Additional file 1: Table S2).

### Sircol assay

Homogenized lungs were analyzed using a Soluble Collagen Assay Kit (BioVision, Milpitas, CA, USA), according to the manufacturer’s instructions.

### Human umbilical cord-derived mesenchymal stromal cells

All procedures involving human umbilical cord MSCs (CTI-195, Cell2in, Seoul, Korea) were performed according to the guidelines of the Seoul National University Hospital Institutional Review Board (IRB No. C-1708-083-878). All MSC isolation procedures are outlined in further detail in the Supplementary Methods section. For the in vivo treatment, 1 × 10^5^ MSCs were administered intratracheally or intravenously on day 10. For the in vitro experiments, 1 × 10^4^ MSCs were seeded into each well.

### Statistical analysis

All statistical analyses were conducted using GraphPad Prism 10 software (GraphPad Software, San Diego, CA, USA). Data are represented as the mean ± standard error of the mean. The Mann–Whitney test was used to compare two groups, and one-way analysis of variance with Tukey’s post-hoc test was used to compare four or more groups. *P*-values < 0.05 were considered to be statistically significant.

## Results

### Increased numbers of macrophage and monocyte markers in terminally fibrotic areas of lungs from patients with IPF

**Fig. 1:**
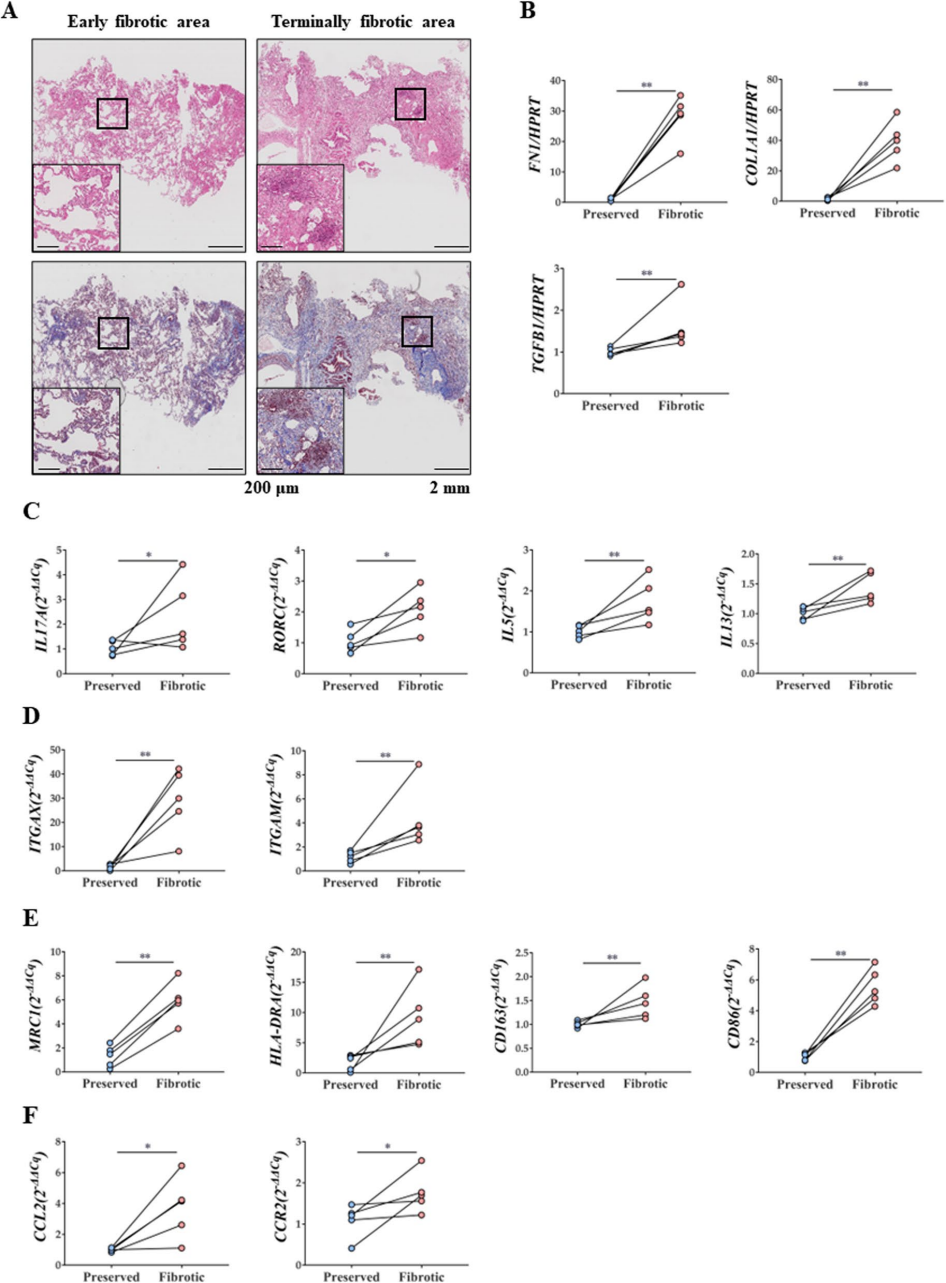
Pathophysiologic changes in samples of patients with IPF. **A** H&E stain and MT stain (× 40 and × 100) of lung tissue of patients with IPF. **B** Changes in *FN1*, *COL1A1*, and *TGB1* mRNA expression in lungs of patients with IPF. **C** Changes in *IL17A, RORC, IL5and IL13* mRNA expression in lungs of patients with IPF. **D** Changes in *ITGAX* and *ITGAM* mRNA expression in lungs of patients with IPF. **E** *MRC1*, * HLA-DRA* *CD163, and CD86* mRNA expression in lungs of patients with IPF. **F** *CCL2* and *CCR2* mRNA expression in lungs of patients with IPF. n = 5 for each group, *indicates P < 0.05, **indicates P < 0.01. All results are representative of at least three independent experiments. IPF idiopathic pulmonary fibrosis, H&E hematoxylin and eosin, MT Masson's trichrome, IL interleukin

We confirmed that the degree of inflammatory cell infiltration and fibrosis in the histological examination was in accordance with the gross findings (Fig. 1A) [44, 45]. Increased expression levels of fibrosis-related markers, such as fibronectin 1 (*FN1*), collagen type 1 alpha 1 chain (*COL1A1*), and *TGF-β1*, were observed in the terminally fibrotic area (Fig. 1B).

Expression levels of *IL17A* and its master regulator, RAR-related orphan receptor C (*RORC*), were elevated in the terminally fibrotic area. In addition, the expression levels of Th2 cytokines were also increased (Fig. 1C). Expression levels of the macrophage surface markers, integrin subunit alpha X (*ITGAX*, known as CD11c) and integrin subunit alpha M (*ITGAM*, known as CD11b), were highly elevated in the terminally fibrotic area(Fig. 1D). Moreover, the expression levels of M1 and M2 macrophage activation-related markers and monocyte chemokines were also increased in the terminally fibrotic area (Fig. 1E, F).

### Increased number of classical monocytes and decreased number of non-classical monocytes in terminally fibrotic areas

**Fig. 2:**
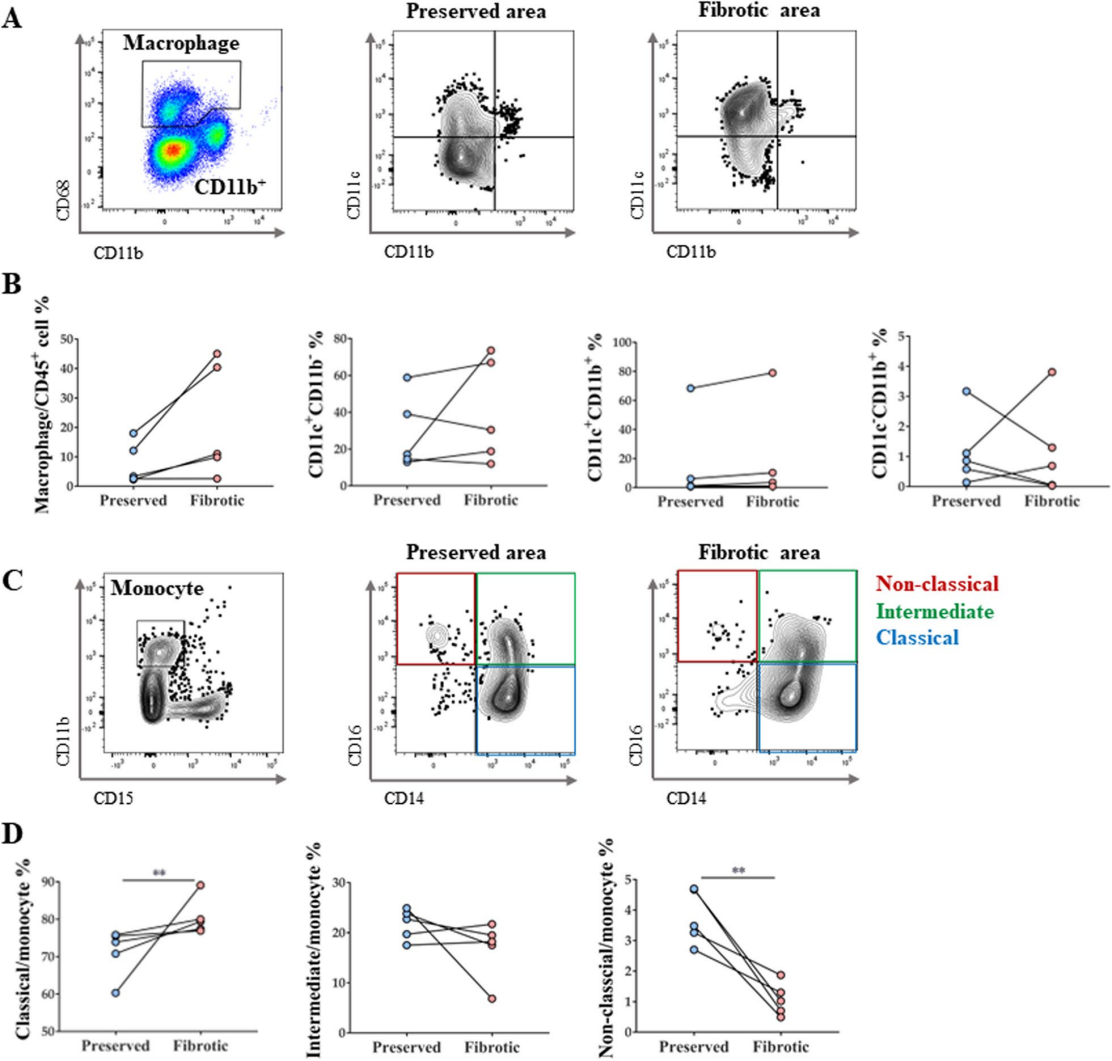
Monocyte and macrophage subtype changes in IPF patient lungs. **A** Macrophage gating protocol and dot plots showing lung AM and MoM distribution in vivo according to CD11c versus CD11b marker expression. **B** Changes in total macrophages and their subtypes in lungs of patients with IPF. **C** Gating protocol of monocytes and their subtypes. **D** Changes in monocyte subtype in lungs of patients with IPF. n = 5 for each group, *indicates P < 0.05, **indicates P < 0.01. All results are representative of at least three independent experiments. IPF idiopathic pulmonary fibrosis, AM alveolar macrophages, MoM monocyte-derived macrophage

Results of flow cytometric analysis of macrophages from explanted human lung tissues are shown in Fig. 2A. In the terminally fibrotic area, the total number of lung macrophages, tended to increase, albeit insignificantly (Fig. 2B). Analyses of monocyte populations (Fig. 2C) revealed a significant increase in the classical monocyte population and a decrease in the non-classical monocyte population (Fig. 2D).

### Differences between macrophage subsets and their fibrotic activities according to their origin

**Fig. 3:**
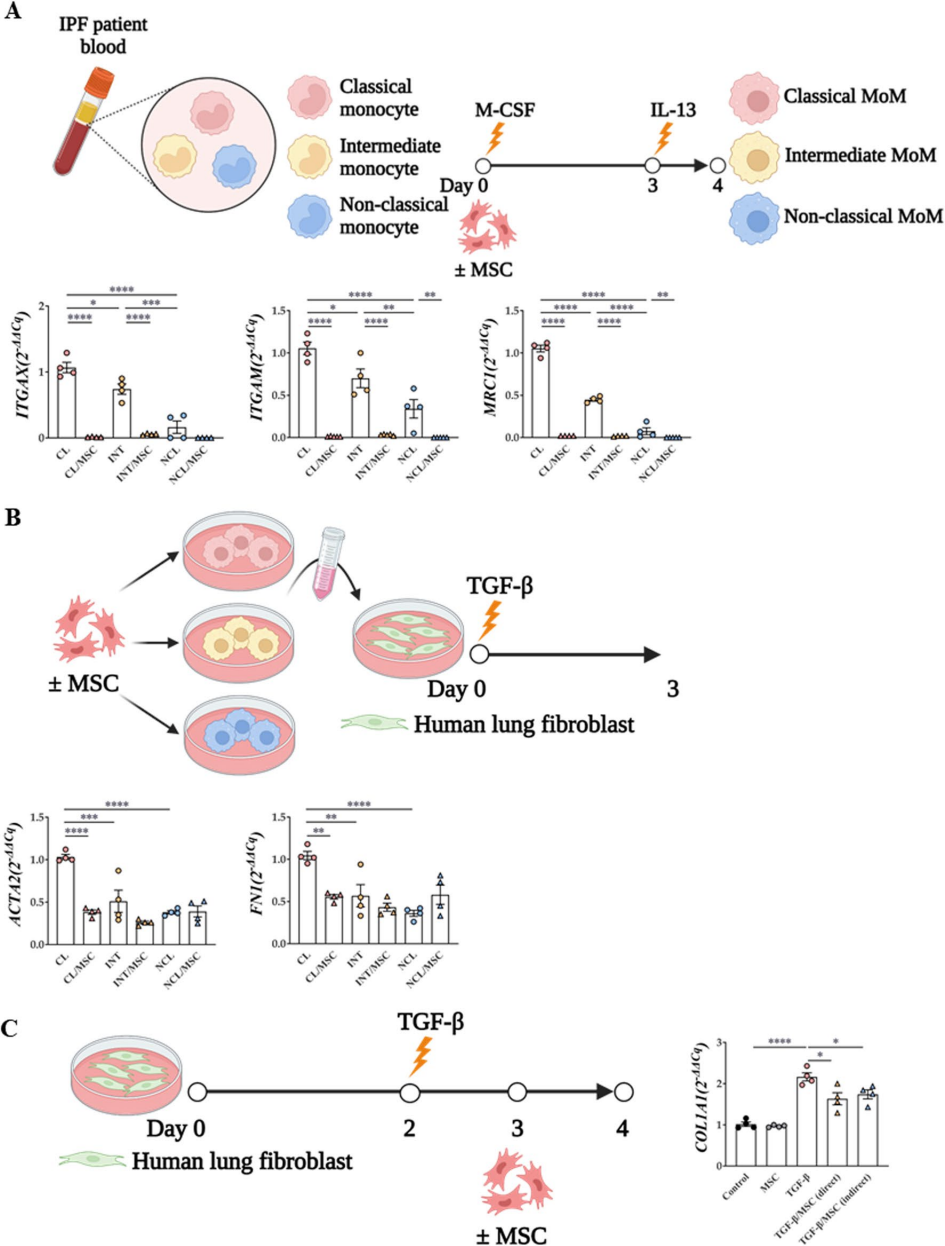
Confirmation of lung fibrosis induction by subtypes of monocyte and macrophage. **A** Monocyte subtype isolation and macrophage differentiation protocol and their macrophage differentiation ability and M2 activation ability **B** Monocyte-derived macrophage culture media transfer protocol and the ability of monocyte subtypes to induce fibrosis. *indicates P < 0.05, **indicates P < 0.01, ***indicates P < 0.001, ****indicates P<0.0001

When human MoMs were stimulated with IL-13, mRNA expression levels of *ITGAX* (CD11c), *ITGAM* (CD11b), and mannose receptor C-type 1 (*MRC1*, also known as CD206) were most frequently elevated in the classical monocyte population (Fig. 3A). This effect was reversed by MSC treatment.

When the supernatant of each monocyte subset was added to the culture medium of human lung fibroblasts (CCL-117), the lung fibrosis-inducing effect was most prominent in the group treated with the supernatant of the macrophages from classical monocytes (Fig. 3B). This fibrotic tendency was alleviated by MSC treatment (Fig. 3A, B). In the CCL-117 in vitro fibrosis model, increased expression levels of *COL1A1* by TGF-β1 were mitigated by MSCs, indicating that MSCs exert anti-fibrotic effects (Fig. 3C).

### Attenuation of inflammation by MSCs in the fibrosis mouse model

**Fig. 4:**
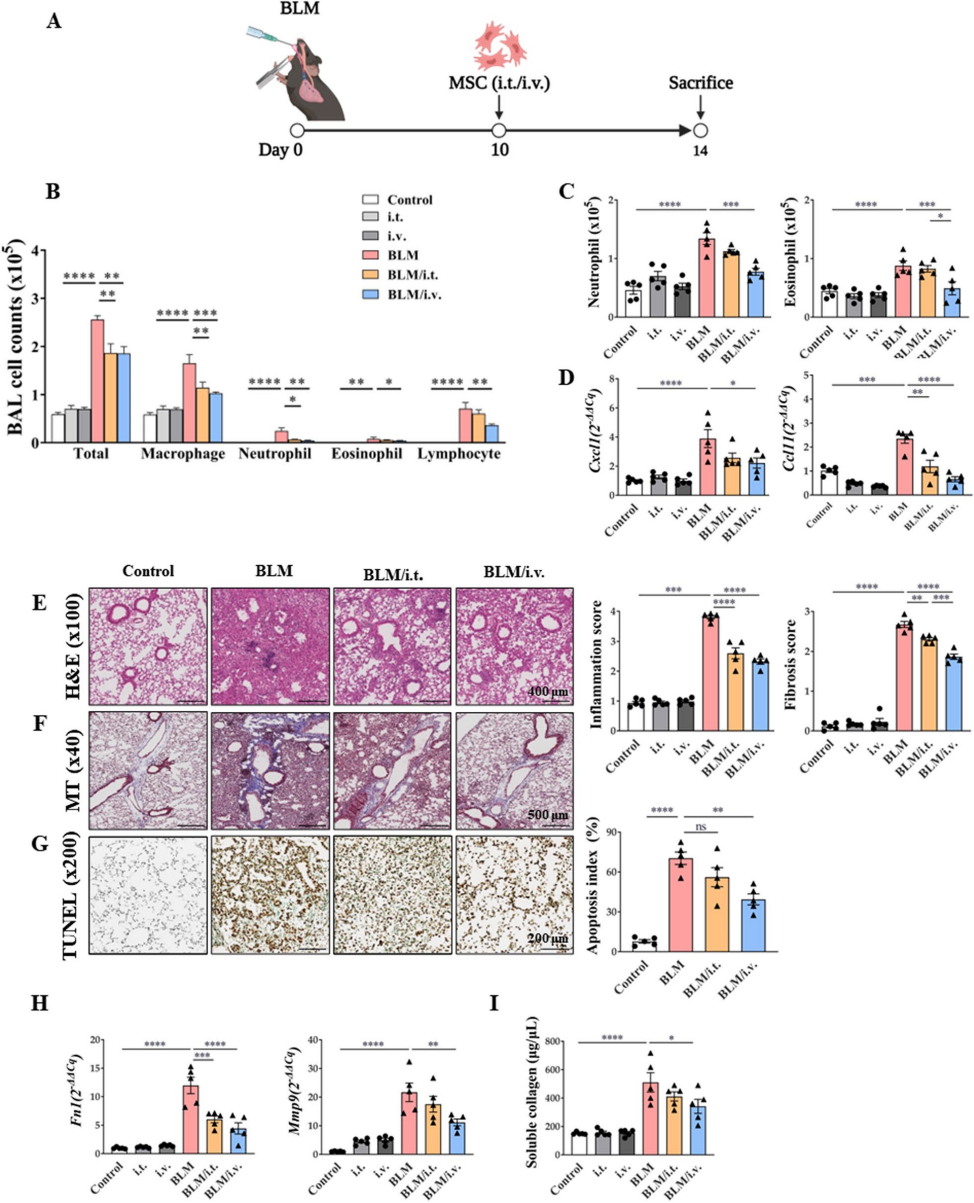
Therapeutic effect of MSCs in a murine fibrosis model on the modulation of exacerbated inflammation. **A** The murine BLM-induced fibrosis model used in the study; administration of intratracheal BLM at 3 mg/kg on day 0. Intratracheal or intravenous treatment of MSCs (10^5^ cells) on day 10. **B** The change of immune cells including macrophages, neutrophils, eosinophils, and lymphocytes in BAL fluid. **C** The number of neutrophils and eosinophils in lungs analyzed by flow cytometry. **D** Chemotaxis mRNA changes associated with neutrophils and eosinophils in the lung. **E** H&E stain (× 100) of lung histology and their scoring; Inflammation score: 0 (absent), 1 (discrete), 2 (mild), 3 (moderate), and 4 (intense). **F** MT stain (× 40) of lung histology and their scoring; Fibrosis score: 0 (none), 1 (mild), 2 (moderate), and 3 (severe). **G** TUNEL stain (× 200) of lung histology and their scoring; Apoptosis index (%): Quantification of the TUNEL-staining cells. **H** *Fn1* and *Mmp9* expression in lungs. **I** Result of Sircol assay showing changes in soluble collagen concentration in the lungs. n = 5 for each group, *indicates *P* < 0.05, **indicates *P* < 0.01, ***indicates *P* < 0.001, ****indicates *P* < 0.0001. All results are representative of at least three independent experiments. MSC mesenchymal stem cell, BLM bleomycin, BAL bronchoalveolar lavage, H&E hematoxylin and eosin, MT Masson's trichrome, TUNEL terminal deoxynucleotidyl transferase dUTP nick-end labeling, *Fn* fibronectin, *Mmp9* matrix metalloproteinase-9

Therapeutic effects of MSCs in the BLM mouse model are shown in Fig. 4. Administration of MSCs significantly lowered the numbers of various inflammatory cells, including macrophages, neutrophils, and eosinophils in BALF (Fig. 4B). While the intravenous injection of MSCs significantly reduced the numbers of neutrophils and eosinophils in the lung tissues, intratracheal administration did not exert the same effect (Fig. 4C). Expression levels of molecules associated with neutrophil and eosinophil chemotaxis were reduced by intravenously injected MSCs (Fig. 4D).

Histological analysis of tissues stained with H&E and MT revealed that MSCs reduced inflammation and fibrosis in the BLM model (Fig. 4E, F) [44, 45]. TUNEL staining revealed that MSCs reduced the degree of apoptosis in lung epithelial cells in the BLM model (Fig. 4G). After MSC administration, a reduction in the number of PAS-positive cells was observed (Additional file 1: Fig. S4) [43]. MSCs also showed reduced mRNA expression levels of *Fn1* and *Mmp9* (Fig. 4H). Finally, intravenously administered MSCs significantly reduced the soluble collagen concentration that was increased by BLM administration (Fig. 4I).

### Modulation of MSCs on lymphoid cells

**Fig. 5:**
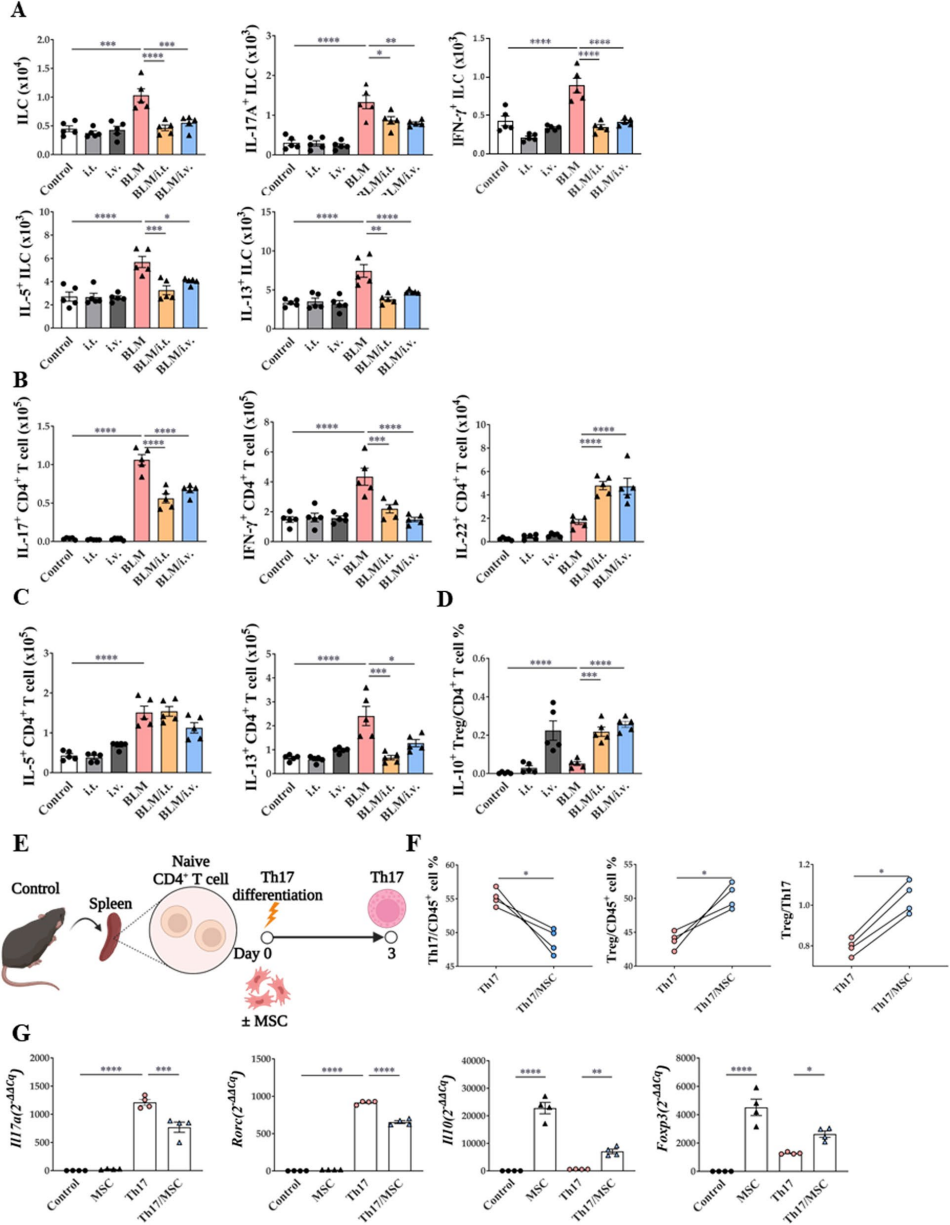
Modulating effect of MSCs on ILCs and T cells activation in a BLM-induced lung fibrosis model. **A** The number of ILCs, IL-17A^+^ ILCs, IFN-γ^+^ ILCs, IL-5^+^ ILCs and IL-13^+^ ILCs in lungs. **B** The number of IL-17A^+^ CD4^+^ T cells, IFN-γ^+^ CD4^+^ T cells, and IL-22^+^ CD4^+^ T cells in lungs. **C** The number of IL-5^+^ CD4^+^ T cells and IL-13^+^ CD4^+^ T cells in lungs. **D** The ratio of IL-10^+^ Tregs in lungs. **E** Th17 differentiation protocol. **F** The ratio of Th17 and Treg. **G** Changes in *Il17*, *Rorc*, *Il10,* and *Foxp3* mRNA expression by MSCs in the Th17 cells. n = 4–5 for each group, *indicates P < 0.05, **indicates P < 0.01, ***indicates P < 0.001, ****indicates P < 0.0001. All results are representative of at least three independent experiments. ILC innate lymphoid cell, IL interleukin, IFN interferon, Th17 T helper 17 cells; Treg, regulatory T cells

Cytokine-secreting cells, including innate lymphoid cells (ILCs) and T cells, were analyzed in the BLM model. Both intravenous and intratracheal MSC treatment reduced total ILCs and all ILC subsets (IL-17A^+^ ILCs, IFN-γ^+^ ILCs, IL-5^+^ ILCs, and IL-13^+^ ILCs) induced by BLM (Fig. 5A). However, their percentage of CD45^+^ cells was not regulated (Additional file 1: Fig. S5A).

In terms of CD4^+^ T cells, IL-17^+^ CD4^+^ T cells and IFN-γ^+^ CD4^+^ T cells upregulated in the BLM model were effectively reduced by MSC treatment, regardless of the route of administration (Fig. 5B). However, in terms of the percentage of CD45^+^ cells, the increased IL-17^+^ CD4^+^ T cells in the BLM model were decreased only by the intravenous route, and the IFN-γ^+^ CD4^+^ was not changed in all groups (Additional file 1: Fig. S5B). Conversely, both intravenous and intrathecal MSC administration increased the number and percentage of IL-22^+^ CD4^+^ T-cells. In addition, MSC treatment reduced the number and percentage of IL-13^+^ CD4^+^ T cells (Fig. 5C, Additional file 1: S5C). Interestingly, the proportion of IL-10^+^ Foxp3^+^CD25^+^CD4^+^ T cells; IL-10^+^ regulatory T cells (Tregs), was remarkably enhanced by MSC treatment (Fig. 5D, Additional file 1: S5D).

When CD4^+^ T cells isolated from the mouse spleen were differentiated into Th17 cells with or without MSCs (Fig. 5E), a reduction in Th17 cells and increase in Tregs was observed with MSC treatment (Fig. 5F). Similarly, a decrease in Th17-related marker expression and increase in Treg-related marker expression were observed after MSC treatment (Fig. 5G).

### Changes in gene expression in the lungs via BLM and MSC treatment

**Fig. 6:**
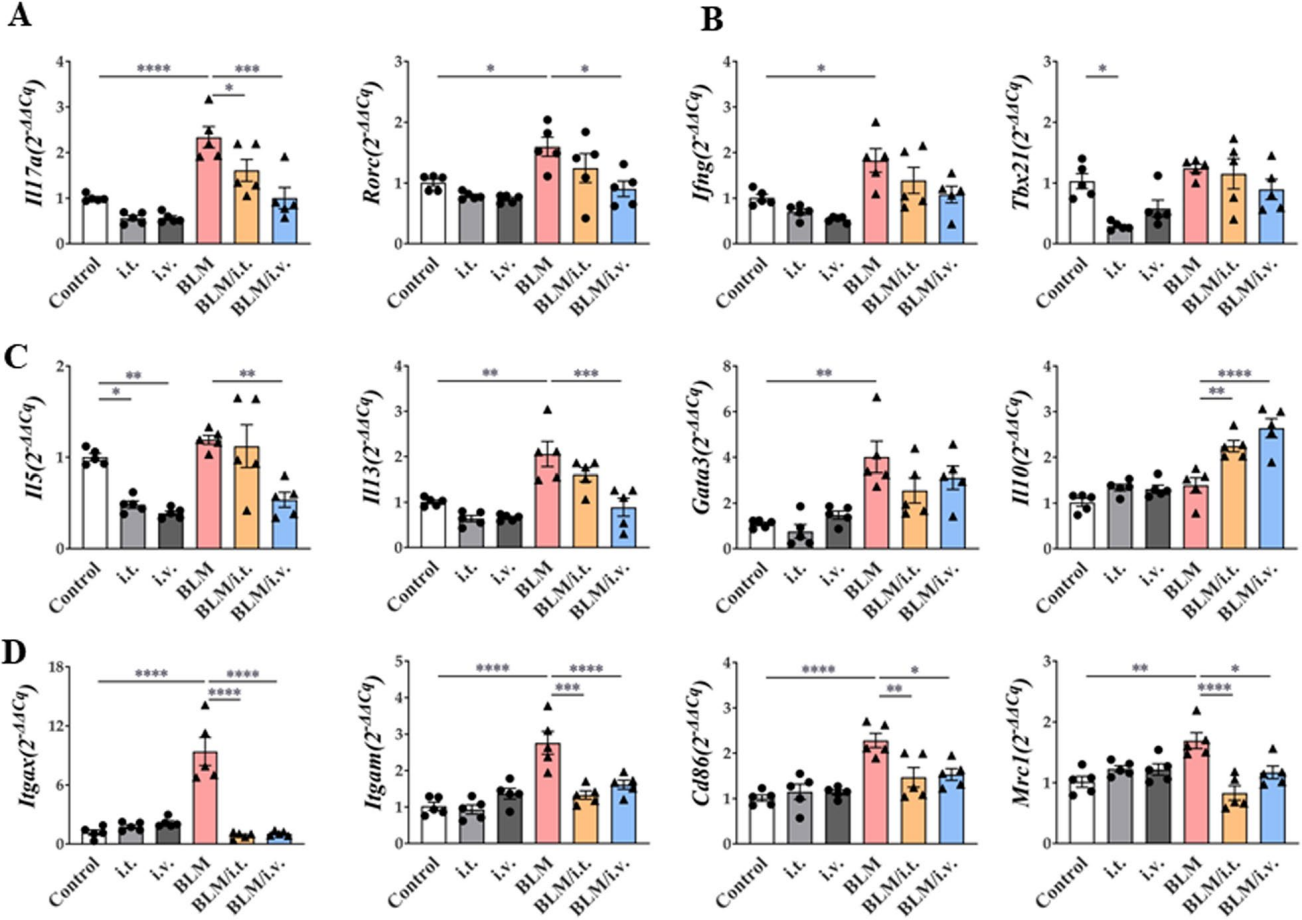
Effect of MSCs on mRNA expression of lung in a murine fibrosis model. **A**–**C** Changes in *Il17a, Rorc, Ifng, Tbx21, Il5, Il13*, *Gata3,* and *Il10* mRNA expression by MSCs in the lungs. **D** Changes in *Itgax, Igtam, Mrc1,* and *Cd86* mRNA expression by MSCs in the lungs. All data were analyzed via real-time quantitative PCR analysis. n = 5 for each group, *indicates *P* < 0.05, **indicates *P* < 0.01, ***indicates *P* < 0.001, ****indicates *P* < 0.0001. All results are representative of at least three independent experiments. *MSC* mesenchymal stem cell, IL interleukin, IFN interferon

Expression levels of *Il17a* and its master regulator, *Rorc*, were increased in the BLM model, and this effect was reversed by the intravenous injection of MSCs (Fig. 6A). Although the expression of *Ifng* was significantly increased in the BLM model, it was not downregulated by MSCs (Fig. 6B). However, there was no significant change in the expression of *T-box transcription factor 21*, the master regulator of Th1 cells, after treatment with BLM or MSCs.

In the BLM model, the mRNA expression levels of *Il5* and *Il13* were effectively downregulated by the intravenous administration, not intratracheal administration, of MSCs. *GATA-binding protein 3* expression was also significantly increased by BLM treatment, but was not affected by MSCs. The expression of inhibitory cytokine *Il10* was significantly enhanced by the intratracheal and intravenous administration of MSCs (Fig. 6C).

Among the markers related to macrophages, increased mRNA expression levels of *Itgax* (CD11c) and *Itgam* (CD11b) were reduced by MSCs in the BLM model. Similarly, the upregulated mRNA expression levels of M1 macrophages (*Cd86*) and M2 macrophages (*Mrc1*) in the BLM model were significantly reduced by MSCs, regardless of the route of administration (Fig. 6D).

### Effect of MSCs on the monocyte/macrophage system in the BLM model

**Fig. 7:**
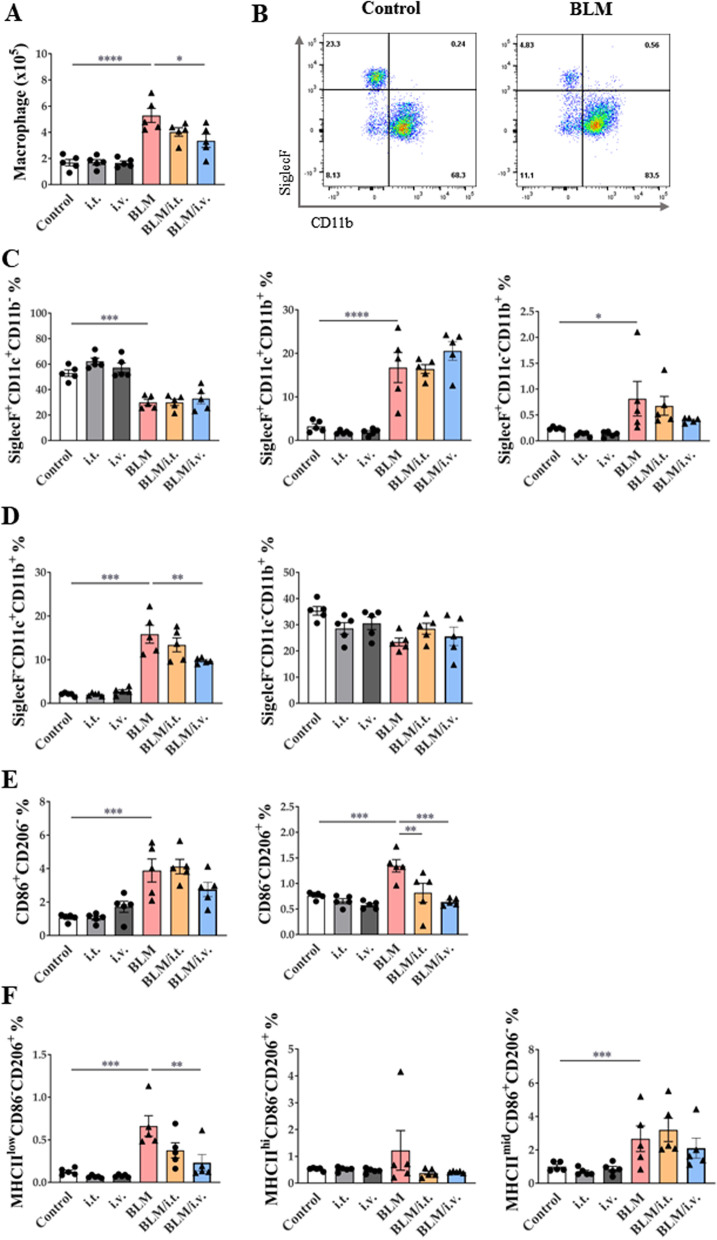
Changes in macrophage subtype activation by MSCs in a murine fibrosis model. **A** The number of lung macrophages. **B** Dot plots showing lung AM and MoM distribution in vivo according to SiglecF versus CD11b marker expression. **C**, **D** Subtypes of SiglecF^+^ and SiglecF^–^ lung macrophages according to CD11c and CD11b expression. **E** Changes in MoMs in the lungs and expression of CD86 and CD206 markers in MoMs (F) Changes in M2 macrophage subpopulations according to the expression of MHCII, CD206, and CD86 markers in the lungs. n = 5 for each group, *indicates *P* < 0.05, **indicates *P* < 0.01, ***indicates *P* < 0.001, ****indicates *P* < 0.0001. All results are representative of at least three independent experiments. MSC mesenchymal stem cell, AM alveolar macrophages, MoM monocyte-derived macrophage

MSCs reduced the number of macrophages in the BLM model (Fig. 7A). Expression levels of CD11b in SiglecF cells were also assessed (Fig. 7B; Additional file 1: Fig. S3B). All SiglecF ^+^ cells expressed CD11c, and most (99.4% in the control group and 81.8% in the BLM group) did not express CD11b. The number of SiglecF^+^CD11c^+^CD11b^–^ macrophages, representative of TR-AMs, was significantly reduced in the BLM model, and MSC administration had no effect on this population. A small number of SiglecF^+^CD11c^+^CD11b^+^macrophages, representative of transitional AMs derived from MoMs, was significantly increased in the BLM group, and MSCs had no effect on this population (Fig. 7C).

Most SiglecF^–^ macrophages were positive for CD11b, and these macrophages were termed as MoMs. Within the SiglecF^–^CD11b^+^ MoM group, the number of SiglecF^–^CD11c^+^CD11b^+^ macrophages was increased in the BLM model, but the intravenous administration of MSCs significantly reduced this subset (Fig. 7D). However, SiglecF^–^CD11c^–^CD11b^+^ macrophages were not altered on days 10 and 4 after BLM and MSC treatment, respectively. Further analysis of SiglecF^–^CD11c^–^CD11b^+^ macrophages according to CD86 and CD206 expression levels was performed to evaluate the changes in the M1 and M2 populations. Although both CD86^+^CD206^–^ and CD86^–^CD206^+^ populations were increased in the BLM model, only the CD86^–^CD206^+^ M2 population was reduced by MSC treatment (Fig. 7E). In terms of the M2 subtype, the number of MHCII^low^CD206^+^CD86^–^ (M2c) macrophages related to fibrosis was increased in the BLM model, and this effects was reversed by intravenously injected MSCs. MSC administration did not significantly affect the MHCII^hi^CD206^+^CD86^–^ (M2a) and MHCII^mid^CD206^–^CD86^+^ (M2b) MoM subtypes (Fig. 7F) [47]. Additional analyses performed on day 21 showed similar results to those on day 14 (Additional file 1: Fig. S6). MSCs also downregulated DCs, including CD11c^+^ DCs and cDC2s, in BLM mice (Additional file 1: Fig. S7).

### Effects of MSCs on macrophages based on their monocyte origin in in vivo and ex vivo models

**Fig. 8:**
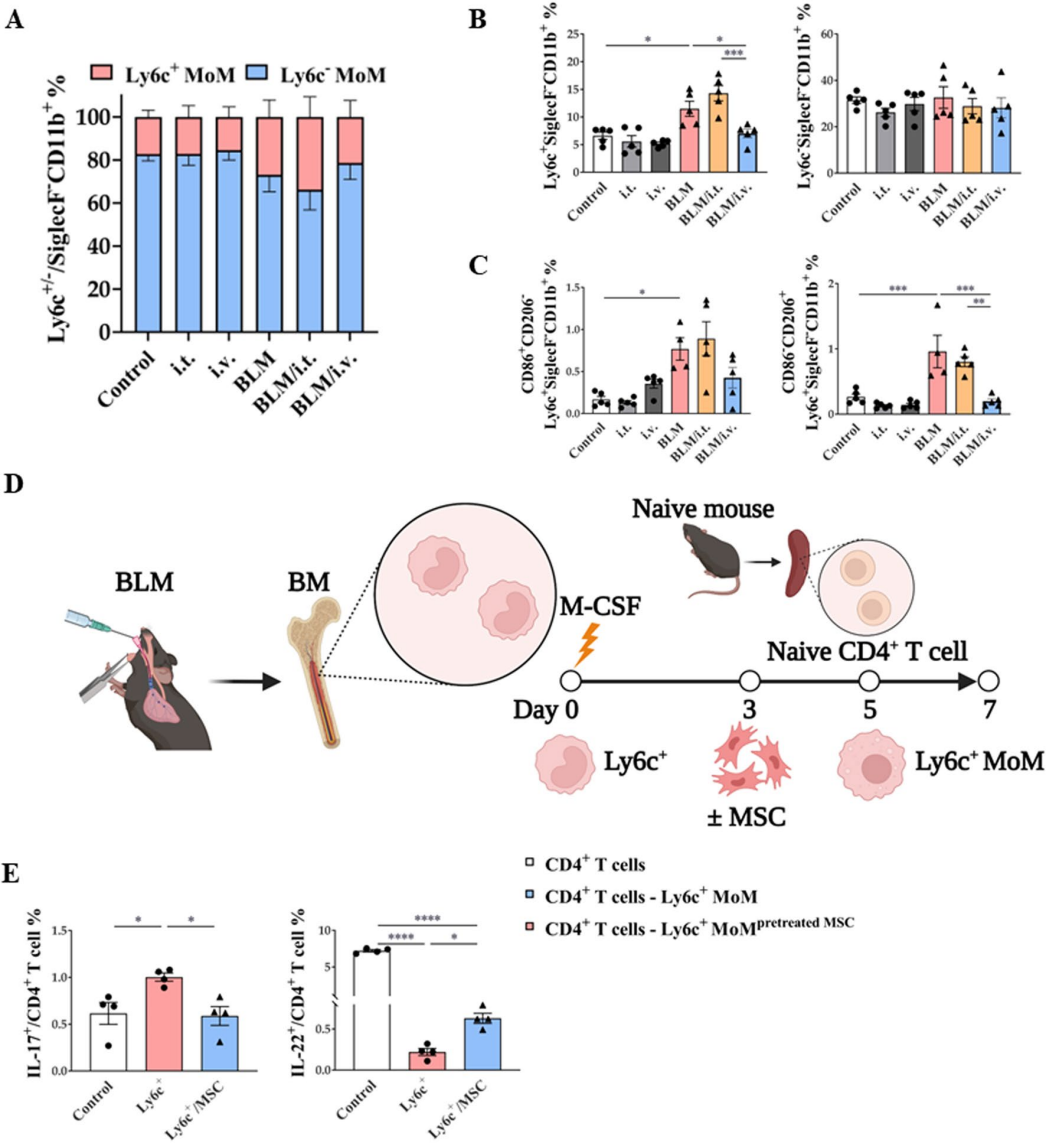
Regulatory effect of MSCs on in vivo and ex vivo Ly6c^+^/^–^ macrophages activation. **A**, **B** Change of Ly6c^+^ and Ly6c^−^ MoMs in lungs. **C** Changes in CD86 and CD206 marker expression populations in Ly6c^+^ MoMs in the lungs. **D** Ex vivo model to evaluate the regulation effect of Ly6c^+^ MoM on CD4^+^ T cell plasticity and the effect of MSC treatment on the regulation **E** Effect of MSC on the plasticity of naive CD4^+^ T cells co-cultured with Ly6c^+^ MoMs. n = 4–5 for each group, *indicates *P* < 0.05, **indicates *P* < 0.01, ***indicates *P* < 0.001, ****indicates *P* < 0.0001. All results are representative of at least three independent experiments. MSC mesenchymal stem cell, MoM monocyte-derived macrophage

To determine their differences based on the origin of MoMs, we classified monocytes into classical and non-classical types based on the surface expression of Ly6c. Ly6c^+^ classical monocytes are recruited to tissues during the initial inflammatory response and differentiate into tissue-specific macrophages. Intravenous MSC administration effectively reduced Ly6c^+^ MoMs in the BLM model, whereas neither BLM nor MSCs showed any significant effects on Ly6c^–^ MoMs (Fig. 8A, B). The main target of the Ly6c^+^ MoM population was the CD86^–^CD206^+^ (M2) population (Fig. 8C).

Next, we established an ex vivo model using bone marrow-derived macrophages to determine the function of Ly6c^+^ MoMs in CD4^+^ T cells and the effect of MSC regulation (Fig. 8D). The plasticity of naive CD4^+^ T cells co-cultured with Ly6c^+^ MoMs was dependent on Th17 differentiation (Fig. 8E). Meanwhile, the ratio of IL-22^+^ CD4^+^ T cells was significantly decreased in the Ly6^+^ MoM co-cultured group compared to that in the control group, and MSC pretreatment with Ly6c^+^ MoMs reversed this effect (Fig. 8E).

### Effects of MSCs on macrophages based on their monocyte origin in ex vivo and in vitro models

**Fig. 9:**
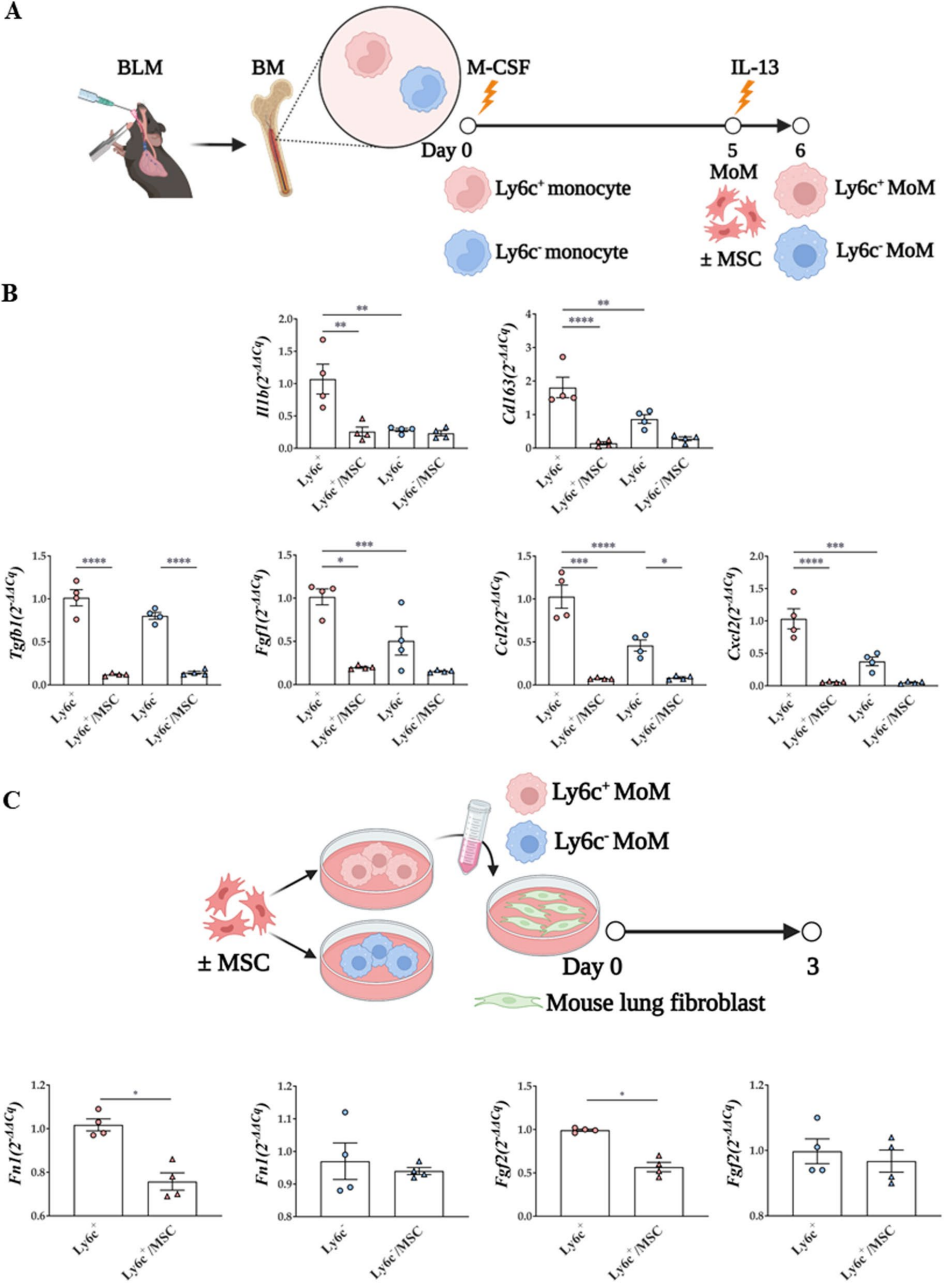
Regulatory effect of MSCs on and ex vivo and in vitro Ly6c^+^/^–^ macrophages activation. **A** Macrophage differentiation protocol of Ly6c^+^ or Ly6c^–^ monocytes isolated from the BLM model. **B** mRNA levels of macrophage activation markers, fibrosis-related markers, and immune cell chemotaxis markers in Ly6c^+^ or Ly6c^–^MoMs. **C** Comparison of fibrosis-inducing ability of Ly6c^+^ or Ly6c^–^ MoMs. n = 4–5 for each group, *indicates *P* < 0.05, **indicates *P* < 0.01, ***indicates *P* < 0.001, ****indicates *P* < 0.0001. All results are representative of at least three independent experiments. MSC mesenchymal stem cell, BLM bleomycin, MoM monocyte-derived macrophage

Comparing Ly6c^+^ or Ly6c^–^ MoMs from BLM-treated mice to assess the fibrogenic ability of MoMs based on their origin (Fig. 9A), *Il1b* (M1 activation marker) and *Cd163* (M2 activation marker) were found to be more highly expressed in Ly6c^+^ MoMs than in Ly6c^–^MoMs, and this effect was diminished by MSC treatment. For monocyte chemotaxis, the levels of C–C motif chemokine ligand 22 (*Ccl22*) and C-X-C motif chemokine ligand 2 (*Cxcl2*) were also significantly increased in MoMs, more prominently in Ly6c^+^ MoMs than in Ly6c^−^MoMs, and this effect was reversed by MSC treatment. MSCs also effectively reduced the enhanced expression levels of *Tgfb1* and *fibroblast growth factor 1* (*Fgf1*) in Ly6c^+^ MoMs (Fig. 9B).

Macrophage polarization was not induced in control mice. However, the upregulated expression levels of *Ccl22*, *Cxcl2*, and *Tgf1b* in IL-13-stimulated Ly6c^+^ MoMs were reduced by MSC treatment (Additional file 1: Fig. S8).

Finally, to evaluate the anti-fibrogenic effects of MSCs on fibroblasts, culture supernatants of Ly6c^+^ and Ly6c^–^ MoMs were transferred to mouse lung fibroblasts (MLg). *Fn1* and *Fgf2* expression levels in the presence of the Ly6c^+^ MoM supernatant were reduced in fibroblasts following MSC treatment. However, these anti-fibrogenic changes were not observed in fibroblasts treated with the Ly6c^–^ MoM supernatant (Fig. 9C).

## Discussion

This study shows that classical monocytes and related MoMs may play an important role in the pathogenesis of IPF and BLM-induced pulmonary fibrosis. The administration of MSCs significantly reduced the number of classical monocyte-derived MoMs, thereby ameliorating the degree of pulmonary fibrosis in a mouse model. In addition, MSC treatment selectively reduced the M2 population, particularly the M2c population. This anti-fibrotic effect was more pronounced when MSCs were administered intravenously.

Various stimuli, such as smoking, pollutant exposure, and gastroesophageal reflux, cause microinjuries that may trigger IPF pathogenesis [48]. The inflammatory immune response elicited by repetitive epithelial cell injury has been investigated in both humans and murine models. Possible immunological mechanisms underlying IPF include aberrant activation of CD4^+^ T cells and adaptive Th2 and Th17 cell responses that induce extracellular matrix production and fibroblast proliferation [49].

Several studies have reported that MoMs play a key role in the pathogenesis of pulmonary fibrosis [50–52]. MoMs exert a paracrine effect by secreting platelet-derived growth factor subunit A, which induces the proliferation of fibroblasts; an autocrine effect on M-CSF is needed to maintain MoMs within fibrotic niches [53, 54]. Circulating monocytes are the main origin of MoMs and are mostly comprised of classical monocytes that infiltrate the lungs via C–C motif chemokine receptor 2 (CCR2). Previous studies have shown that depletion of circulating monocytes or CCR2 deficiency significantly reduces the degree of pulmonary fibrosis, suggesting that MoMs derived from classical monocytes may play a critical role in the development of pulmonary fibrosis [55–60]. In contrast, AMs play important roles in the maintenance of homeostasis, clearance, and immune regulation in healthy lungs. In fact, the number of SiglecF^+^CD11c^+^CD11b^–^ macrophages representing TR-AMs was significantly decreased by intratracheal BLM instillation, which is consistent with the findings of a previous study [50].

We divided MoMs into classical and non-classical MoMs using Ly6c expression as a marker of classical monocytes and found that BLM administration significantly increased the number of classical MoMs. These findings are consistent with those of a human study that reported increased numbers of classical monocytes in the fibrotic areas of the lungs and blood of patients with IPF [50]. Another study reported the expansion of lung macrophages derived from monocytes in explanted lungs of recipients with pulmonary fibrosis [50]. Transcriptome analysis revealed that 61 genes out of all human homologs of the mouse pro-fibrotic genes identified in the fibrosis model were differentially expressed between lung macrophages isolated from recipients and donors, indicating the possibility of common pro-fibrotic pathways in mouse and human macrophages [50]. In our study, we also observed a significant increase in the expression levels of macrophage-related genes (*ITGAM, ITGAX, MRC1, CD86*, and *HLA-DRA*) and monocyte chemotaxis genes (*CCL2* and *CCR2)* in the terminally fibrotic areas of the lungs of patients with IPF. In addition, flow cytometry analysis revealed an increase in classical monocyte population and decrease in non-classical monocyte population in the terminally fibrotic area.

In terms of plasticity in vivo, M1/M2 polarization is unlikely to occur in vivo as observed in vitro. However, for the functional phenotype of macrophages, studies have reported that deletion of the M2-associated gene inhibits M2 polarization and protects against BLM-induced pulmonary fibrosis and that administration of M2 restores the susceptibility to BLM [61, 62]. In addition, a study evaluating BALF of patients with acute exacerbations of IPF showed an upregulation in the levels of M2 cytokines, including IL-1 receptor antagonist and CCL2 [63]. In our study, flow cytometry revealed an increase in both CD86^+^CD206^–^ (M1) and CD86^–^CD206^+^ (M2) MoM populations in the BLM group. However, further investigations are needed to understand the M1/M2 dynamics in response to lung injury.

In addition to macrophages, immune cells, including T cells, granulocytes, and ILCs, are involved in the pathogenesis of pulmonary fibrosis. Recent studies have shown that Th1, Th2, Th9, Th17, and T follicular helper cells exert pro-fibrotic effects, whereas Th22 cells exhibit anti-fibrotic effects [64]. Tregs are considered to possess both pro-fibrotic and antifibrotic properties [65]. In the early stages of inflammation, Tregs can exacerbate the inflammatory response, but in the late stages, they hinder the development of fibrosis [66]. ILCs, a family of innate immune cells that mirror T cells [67], have been extensively studied in recent years. Many studies have reported that ILC2 and ILC3 may induce fibrosis via the release of various cytokines [68–71].

MSCs regulate pulmonary fibrosis via direct cell–cell interactions and secretion of bioactive molecules, including growth factors and EVs [72]. MSCs regulate diverse immune cells and have been reported to reduce the number of inflammatory cells in the airways [31, 32, 73]. MSCs have also been shown to decrease inflammatory cell infiltration in the lung tissues in mouse models of fibrosis [31, 32, 74]. These findings are consistent with ours in this study, as MSCs significantly decreased the proportions of all inflammatory cells in the BALF of the BLM mouse model. Furthermore, our study demonstrated that several of the augmented Th responses in the BLM group were significantly reduced by MSC treatment, and MSCs restored the Th22 cells decreased in the BLM group. We also demonstrated that MSCs significantly decreased not only the total ILC count but also the counts of ILC1s, ILC2s, and ILC3s in the BLM group. To date, only a few studies have reported the effects of MSCs on ILCs, mainly ILC2 [75, 76].

Macrophages are another major target of MSCs via immune modulation [77]. In our study, the intravenous administration of MSCs decreased the total number of macrophages in the BLM group. However, MSC treatment did not have any significant effect on TR-AMs or macrophages with newly acquired AM characteristics. Although SiglecF^–^CD11c^–^CD11b^+^ macrophages, reflecting MoMs, showed no significant changes following MSC administration, intravenous MSCs significantly reduced the number of M2 MoMs in the BLM group. To our knowledge, this study is the first to report the effects of intravenously administered MSCs on the number of M2c macrophages. Among the M2 subtypes, M2c has been reported to secrete TGF-β and IL-10 to suppress inflammation, repair damaged areas, participate in tissue remodeling, and prevent fibrosis [78]. Many studies have focused on the anti-inflammatory effects of MSCs on lung injury, and administration of MSCs in the early stages of lung injury has been suggested to prevent pulmonary fibrosis via immune modulation [79]. Recent studies have shown that MSCs can reverse fully established pulmonary fibrosis up to 21 or 28 d after BLM administration in rats [80, 81].

Murine monocytes can be divided according to their surface expression of Ly6c. Ly6c^+^ monocytes are recruited to tissues on demand and eventually differentiate into tissue-specific macrophages capable of self-renewal [82]. We observed that the increased number of Ly6c^+^ MoMs in the BLM group was significantly reduced by the intravenous administration of MSCs. In ex vivo experiments using Ly6c^+^ or Ly6c^–^ monocytes from the bone marrow of the BLM and control groups, the effects of MSCs were more pronounced for Ly6c^+^ MoMs, indicating that the therapeutic effects of MSCs were more evident in the presence of damage or inflammation rather than in a state of constant homeostasis. In addition, Ly6c^+^ MoMs exerted regulatory effects on the plasticity of naïve CD4^+^ T cells. Therefore, the induction of differentiation of naïve CD4^+^ T cells into IL-17^+^ CD4^+^ T cells by Ly6c^+^ MoMs may be a potential mechanism for pulmonary fibrosis. Our results suggest that the mechanism underlying the anti-fibrotic effect of MSCs may involve the regulation of Th17 and Th22 cells.

There are two main delivery routes for MSCs, intravenous and intratracheal administration, which are the most commonly used routes for the treatment of pulmonary diseases. Intravenous administration was used in 87.5% of studies on pulmonary fibrosis [83]. According to the literature, a substantial amount of intravenously administered MSCs remains trapped in the lungs during the first transfer [84], and most MSCs are observed in the lungs 5 min after administration in a mouse model [85]. However, previous reports on more effective routes of administration have shown conflicting results [86–89]. In this study, we compared the effectiveness of intravenous and intratracheal administration and found that intravenous administration of MSCs was more effective in reducing lung inflammation and fibrosis than intratracheal administration. This finding can be explained by the fact that intravenous administration enables more rapid and effective activation of circulating monocytes compared to intratracheal administration. A previous study showed that the transfer of preconditioned bone marrow-derived monocytes with EVs of MSCs significantly improved fibrosis in a BLM-induced pulmonary fibrosis mouse model, indicating monocytes as one of the main targets of MSCs [34].

This study has several limitations. First, macrophage composition and phenotypes in the lungs exhibit considerable time-dependent variations that limit the generalizability of experiments on pulmonary fibrosis models at a certain time point. Therefore, we analyzed the mouse model on days 14 and 21 and confirmed that the results were consistent over time. Second, the exact subtypes of macrophages were not verified in this study, although representative cellular markers were used to distinguish between the subtypes. We did not use knockout mice of each macrophage subtype to confirm whether each macrophage subtype directly affected the development of pulmonary fibrosis; hence, this should be further explored in future studies. Third, the specific cause for the decrease in the expression levels of fibrosis-related genes in lung fibroblasts after the addition of media with the MSC and MoM co-culture remains unknown. Exosomes secreted by MSCs may possibly be involved in this process [34]. Lastly, our results implied the contribution of specific cells on the effects of MSCs based on the verified effects of MSCs, especially each macrophage subtype, on different immune cells. However, the underlying signaling pathways and drug-mediated intervention mechanisms remain unclear and require further elucidation in future studies.

## Conclusions

In conclusion, our results indicate that both classical monocytes and MoMs play important roles in the development of pulmonary fibrosis. Treatment with intravenous MSCs can reduce pulmonary fibrosis by inhibiting M2 activation of MoMs and modulating classical monocytes.

## Supplementary Information

**Additional file 1.** Methods and materials. **Fig. S1.** Gating strategy for human macrophage and monocyte. **Fig. S2**. Gating strategy for murine T cells and ILC. **Fig. S3.** Gating strategy for murine macrophages. **Fig. S4.** PAS staining analysis of lungs in a murine fibrosis model. **Fig. S5.** Modulating effect of MSCs on ILCs and T cells ratio in a BLM-induced lung fibrosis model. **Fig. S6.** Changes in macrophage subtype activation by MSCs in a murine fibrosis model on day 21. **Fig. S7.** Effect of MSCs on the activation of subtypes of DCs in a murine fibrosis model. **Fig. S8.** Regulatory effect of MSC on activation of control-derived Ly6c^+^ or Ly6c^-^ macrophages ex vivo. **Table S1.** Flow cytometry antibody list. **Table S2.** Primer sequences used in qPCR amplification

## Data Availability

The datasets used and/or analysed during the current study are available from the corresponding author on reasonable request.

## Abbreviations

IPF: Idiopathic pulmonary fibrosis
MSC: Mesenchymal stem cell
BLM: Bleomycin
MoM: Monocyte-derived macrophage
M2: Type 2 macrophage
AM: Alveolar macrophage
TR-AM: Tissue-resident alveolar macrophage
M1: Type 1 macrophage
IL: Interleukin
IFN: Interferon
TNF: Tumor necrosis factor
TGF: Transforming growth factor
H&E: Hematoxylin and eosin
MT: Masson's trichrome
PBS: Phosphate buffered saline
BALF: Bronchoalveolar lavage fluid
TUNEL: Terminal deoxynucleotidyl transferase dUTP nick end labeling
PAS: Periodic acid-Schiff
RBC: Red blood cell
PBMC: Peripheral blood mononuclear cell
M-CSF: Macrophage colony stimulating factor
RT-qPCR: Quantitative reverse transcription polymerase chain reaction
cDNA: Complementary DNA
hUC: Human umbilical cord
FN1: Fibronectin 1
COL1A1: Collagen type 1 alpha 1 chain
TGFB1: Transforming growth factor beta 1
RORC: RAR-related orphan receptor C
Th: T helper cell
ITGAX: Integrin subunit alpha X
ITGAM: Integrin subunit alpha M
Mmp9: Matrix metallopeptidase 9
ILC: Innate lymphoid cell
Treg: Regulatory T cell
Ifng: Interferon gamma
Tbx21: T-box transcription factor 21
Gata3: GATA binding protein 3
Ccl22: C-C motif chemokine ligand 22
Cxcl2: C-X-C motif chemokine ligand 2
Fgf: Fibroblast growth factor
CCR2: C-C Motif Chemokine Receptor 2
EV: Extracellular vesicle
