# Supplementary material for: Classical monocyte-derived macrophages as therapeutic targets of umbilical cord mesenchymal stem cells: comparison of intratracheal and intravenous administration in a mouse model of pulmonary fibrosis
Source: Respir Res. 2023 Mar 5;24:68. doi: 10.1186/s12931-023-02357-x (PMC9985859; doi:10.1186/s12931-023-02357-x)
Supplement: Supplementary file 1 — Additional file 1. Methods and materials. Fig. S1. Gating strategy for human macrophage and monocyte. Fig. S2. Gating strategy for murine T cells and ILC. Fig. S3. Gating strategy for murine macrophages. Fig. S4. PAS staining analysis of lungs in a murine fibrosis model. Fig. S5. Modulating effect of MSCs on ILCs and T cells ratio in a BLM-induced lung fibrosis model. Fig. S6. Changes in macrophage subtype activation by MSCs in a murine fibrosis model on day 21. Fig. S7. Effect of MSCs on the activation of subtypes of DCs in a murine fibrosis model. Fig. S8. Regulatory effect of MSC on activation of control-derived Ly6c+ or Ly6c- macrophages ex vivo. Table S1. Flow cytometry antibody list. Table S2. Primer sequences used in qPCR amplification [file 12931_2023_2357_MOESM1_ESM.docx]

# Additional file 1: Methods and materials

**Preparation of human umbilical cord-derived mesenchymal stem cells**

The hUC tissues were obtained immediately after full-term births following cesarean section, with informed consent. The hUCs were washed with phosphate-buffered saline (PBS) to remove vessels and amnion. Wharton’s jelly (WJ) tissues within the hUC were isolated and minced. These explants were digested for 3h at 37 °C with an enzyme mixture (Miltenyi Biotec, Bergisch Gladbach, NRW, Germany), filtered through a 100-μm cell strainer (BD Biosciences), and pelleted by low-speed centrifugation at 200 × g for 10 min. The isolated WJ-MSCs were cultured in a CellCor™ CD medium (Xcell Therapeutics, Seoul, Korea) supplemented with 2% human platelet lysates (StemCell Technologies, Vancouver, BC, Canada) in a 37℃ incubator under humidified conditions with 5% CO2. The cells were harvested once they reached 90% confluence. High levels of glutathione (GSH) in hUC-MSCs were isolated from cultured hUC-MSCs using a fluorescent real-time thiol tracer, which is a ratiometric probe capable of monitoring reactive oxygen species-induced GSH changes in living stem cells (1). The cryopreserved hUC-MSCs at passage 4 were cultured up to passage 7 and then used in the experiment (2).

**Cell analysis by flow cytometry**

For human lung single-cell macrophage gating, after determining the population of CD45^+^ cells, macrophages were determined by dividing them into CD68 versus CD11b. Gated macrophages were further analyzed based on CD11c versus CD11b (Fig. S1a). Gated monocytes were divided into CD16 versus CD14 to differentiate subtypes (Fig. S1b).

For mouse lung single-cell CD4^+^ T cell and ILC gating, CD4^+^ T cells were first determined by dividing the determined CD45^+^ cells by lineage versus CD4. The determined CD4^+^ T cells were analyzed separately for each cytokine expression into Th1, Th2 and Th17 cells. Separately, the CD90.2 positive group was determined as ILCs using the remaining groups except for CD4^+^ T cells determined in the group gated by Lineage versus CD4. The determined ILCs were also divided according to the expression of each cytokine, the same as the Th cell subtype (Fig. S2a).

For mouse Treg gating, CD45^+^ cells were equally gated and CD4^+^ cells were determined by dividing CD8 versus CD4 based on CD3^+^ cells. Tregs were gated by dividing by Foxp3 versus CD25 based on CD3^+^CD4^+^ T cells. Gated Tregs were further analyzed for IL-10 cytokine expression.

For mouse eosinophil, DC and macrophage analysis, CD45^+^ cells were identically determined. The determined CD45^+^ cells were divided by CD11c versus SiglecF to determine eosinophils. Populations except for the determined eosinophils were then gated on DCs with F4/80 versus CD11c and macrophages with F4/80 versus CD11b. Gated DCs were divided by MHCII versus CD11b. Then, after determining CD11b^+^ DC, the Ly6c^-^ population was defined as cDC2.

Gated macrophages were divided by SiglecF versus CD11b to determine AM, SiglecF^+^CD11b^+^ macrophage populations and MoM. The determined AM was again analyzed for the CD11c^+^ population. The SiglecF^+^CD11b^+^ macrophage population was further subdivided by CD11c expression for further analysis. SiglecF^-^CD11b^+^CD11c^-^ MoM was determined by dividing it into non-classical or classical according to Ly6c expression.

Separately, the M1, M2, M2a, M2b, and M2c MoM according to the expression of CD206, CD86 and MHCII were further analyzed. all analyzes were determined using the LSR Fortessa X-20 (BD Biosciences) and FlowJo10 software (TreeStar, USA).

**Additional file 1: figures**

# Fig. S1. Gating strategy for human macrophage and monocyte

(a) Only live cell populations were selected using SSC and FSC. Among the selected cells, CD45^+^ cells were gated and the macrophage population was determined using CD68 and CD11b markers. The determined macrophages were divided into subgroups using CD11c and CD11b.

(b) CD45^+^ cells were determined in the same way as macrophages. The monocyte population was then gated using the CD11b and CD15 markers. Gated monocytes were classified into subtypes using CD16 and CD14 markers.


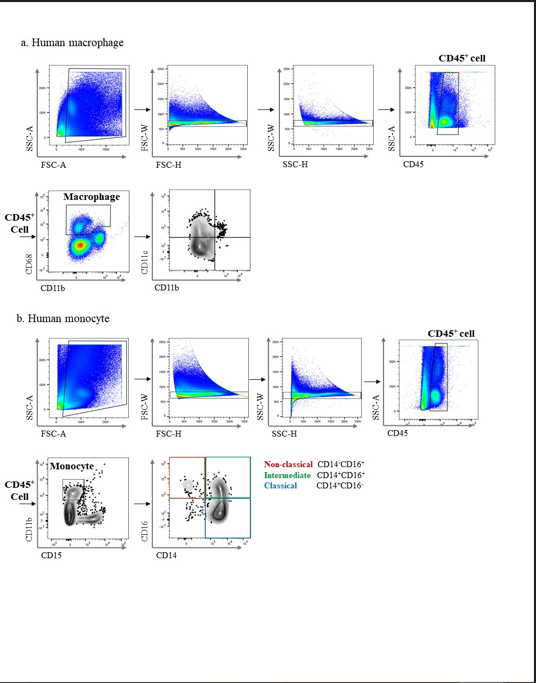


# Fig. S2. Gating strategy for murine T cells and ILC

(a) Live cells were gated from lung single cells using SSC and FSC. After gating live cells, among live cells, lymphocyte population was defined as CD45^+^ cells. Then, CD4^+^ T cells were defined as from lineage versus CD4 scatter plots and ILC were defined from lineage versus CD90.2 scatter plots. Next, subclassify CD4^+^ T cells and ILCs according to cytokines including IL-5, IL-13, IL-17, and IFN-γ.

(b) Live cells were gated from lung single cells using SSC and FSC. After gating live cells, among live cells, lymphocyte population was defined as CD45^+^ cells. Then, gated CD45^+^ cells were subclassed into total T cells using the CD3 marker. Total T cells were subdivided into CD4^+^ T cells and CD8^+^ T cells. Then, Foxp3^+^CD25^+^ cells from the CD4^+^ cells of CD4 versus CD8 scatter plots were defined as Tregs. The cytokine secreting cell gating was determined using fluorescence minus one controls. SSC, side scatter; FSC, forward scatter; ILC, innate lymphoid cells; IL, interleukin; IFN, interferon; Treg, regulatory T cells


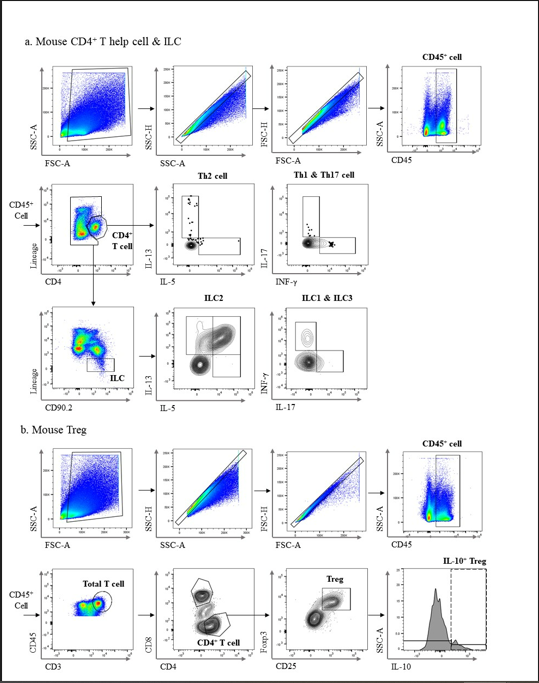


# Fig. S3. Gating strategy for murine macrophages

Lymphocyte population was determined in the same way as T cell and ILC gating. In the CD11c versus SiglecF scatter plot, eosinophils were gated out. Then, after excluding eosinophils, DCs and macrophages were classified using F4/80, CD11c, and CD11b markers. DCs were subdivided into CD11b^+^ DCs and cDC2s with MHCII, CD11b and Ly6c. CD11c and CD11b markers were used to classify AM and MDM in macrophages. In AM, SiglecF expression was further subdivided into RAM. In MDM, several subtypes were divided and analyzed using CD11c and SiglecF markers. Especially, for more precise analysis of MDM, Ly6c, CD11c, CD86 and CD206 markers were used. M1 MDM was defined by gating on CD86^+^CD206^-^CD11c^+^ MDMs. Contrarily, M2 MDM was defined by gating on CD86^-^CD206^+^CD11c^-^ MDM. In addition, M2 subtype was defined as follows. MHCII^hi^CD206^+^CD86^-^ cells, MHCII^low^CD206^+^CD86^-^ cells and MHCII^mid^CD206^-^CD86^+^ cells from CD206 and CD86 histogram plot were classified as M2a, M2b, and M2c, respectively. All macrophage subtypes represent the ratio to total macrophage. Macrophage subtypes gating was determined using fluorescence minus one controls. ILC, innate lymphoid cells; SSC, side scatter; FSC, forward scatter; DC, dendritic cells; cDC2, type 2 conventional DCs; AM, alveolar macrophages; MDM, monocyte-derived macrophage


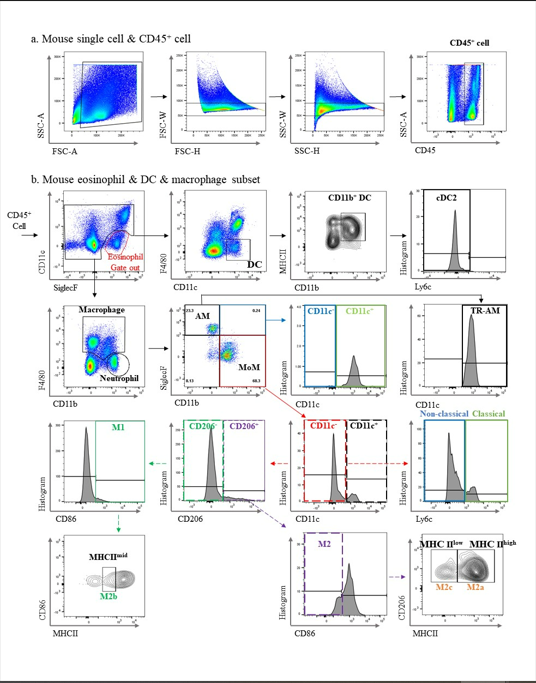


# Fig. S4. PAS staining analysis of lungs in a murine fibrosis model.

PAS staining of lung histology (×100) and its scoring. n=5 for each group, * indicates *P* < 0.05, ** indicates *P* < 0.01, *** indicates *P* < 0.001, **** indicates *P* < 0.0001. All results were representative of at least three independent experiments. PAS, Periodic acid–Schiff


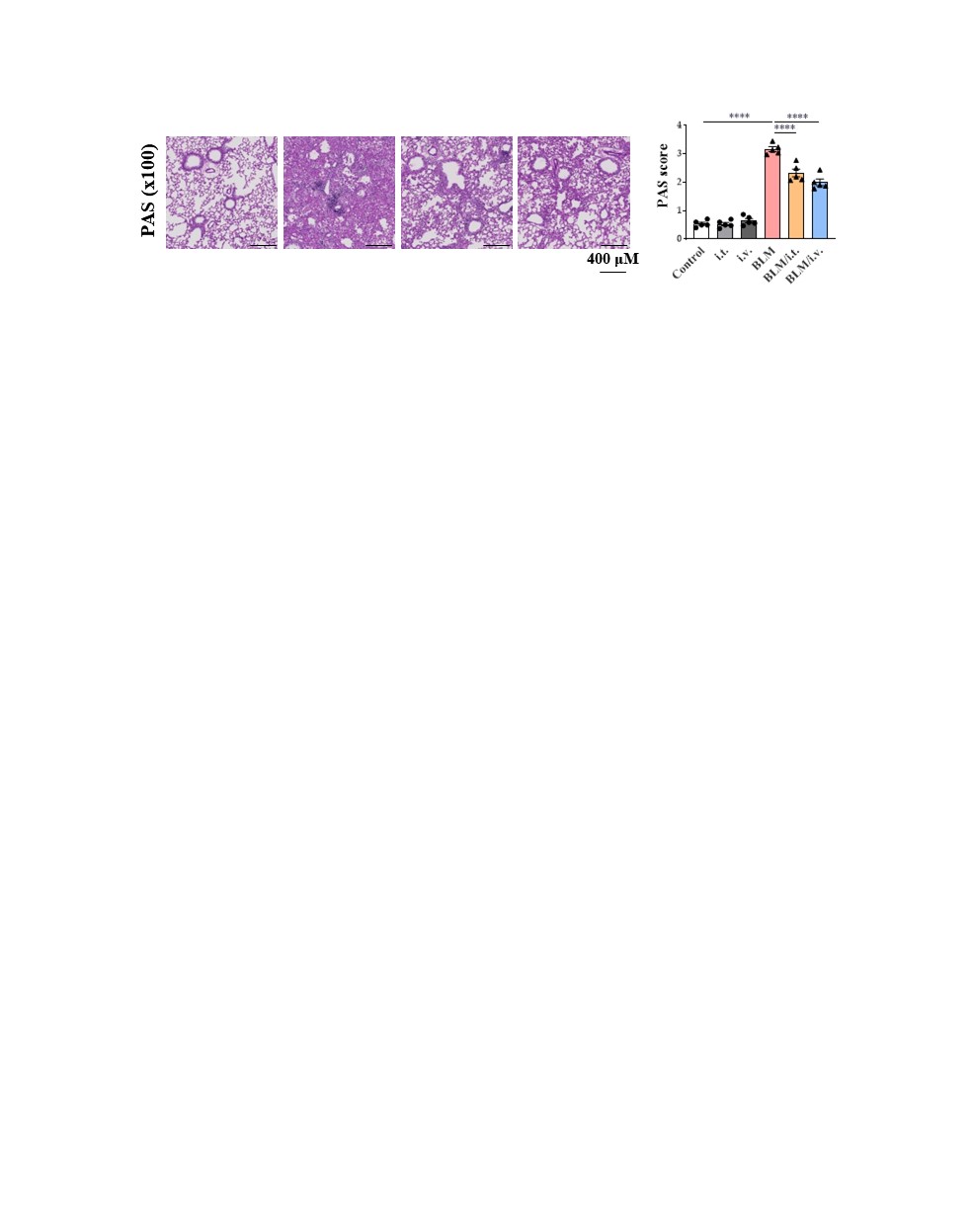


# Fig. S5. Modulating effect of MSCs on ILCs and T cells ratio in a BLM-induced lung fibrosis model

(A) The ratio of ILCs, IL-17^+^ ILCs, IFN-γ^+^ ILCs, IL-5^+^ ILCs and IL-13^+^ ILCs in lungs. (B) The ratio of IL-17^+^ CD4^+^ T cells, IFN-γ^+^ CD4^+^ T cells and IL-22^+^ CD4^+^ T cells in lungs. (C) The ratio of IL-5^+^ CD4^+^ T cells and IL-13^+^ CD4^+^ T cells in lungs. (D) The ratio of IL-10^+^ Foxp3^+^CD25^+^CD4^+^ T cells in lungs. All ratios were expressed as percentages of CD45^+^ cells. n=5 for each group, * indicates *P* < 0.05, ** indicates *P* < 0.01, *** indicates *P* < 0.001, **** indicates *P* < 0.0001. All results are representative of at least three independent experiments. ILC, innate lymphoid cells; IL, interleukin; IFN, interferon; Treg, regulatory T cells


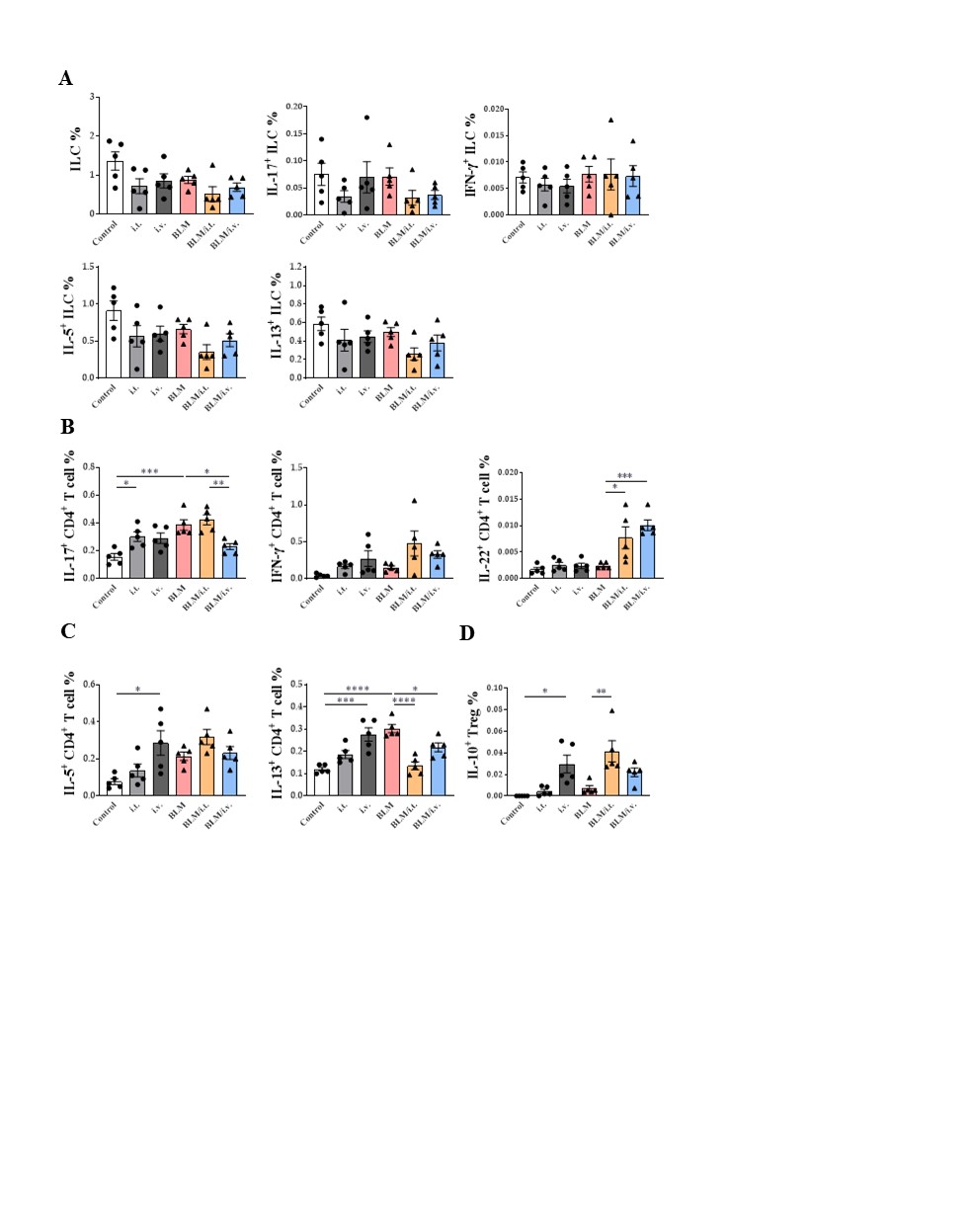


# Fig. S6. Changes in macrophage subtype activation by MSCs in a murine fibrosis model on day 21

(A) The murine BLM-induced fibrosis model on day 21 used in the study; administration of intratracheal BLM at 3 mg/kg on day 0. Intratracheal or intravenous treatment of MSCs (10^5^ cells) on day 10. (B) The number of lung macrophages. (C) Dot plots showing lung AM and MoM distribution *in vivo* according to SiglecF versus CD11b marker expression. (D, E) Subtypes of SiglecF^+^ and SiglecF^-^ lung macrophages according to CD11c and CD11b expression. (F) Changes in MoM in the lungs and expression of CD86 and CD206 markers in MoM (G) Changes in M2 macrophage subpopulations according to the expression of MHCII, CD206, and CD86 markers in the lungs. n =5 for each group, * indicates *P* < 0.05, ** indicates *P* < 0.01, *** indicates *P* < 0.001, **** indicates *P* < 0.0001. All results are representative of at least three independent experiments. BLM, bleomycin; AM, alveolar macrophages; MoM, monocyte-derived macrophage; MSC, mesenchymal stem cell


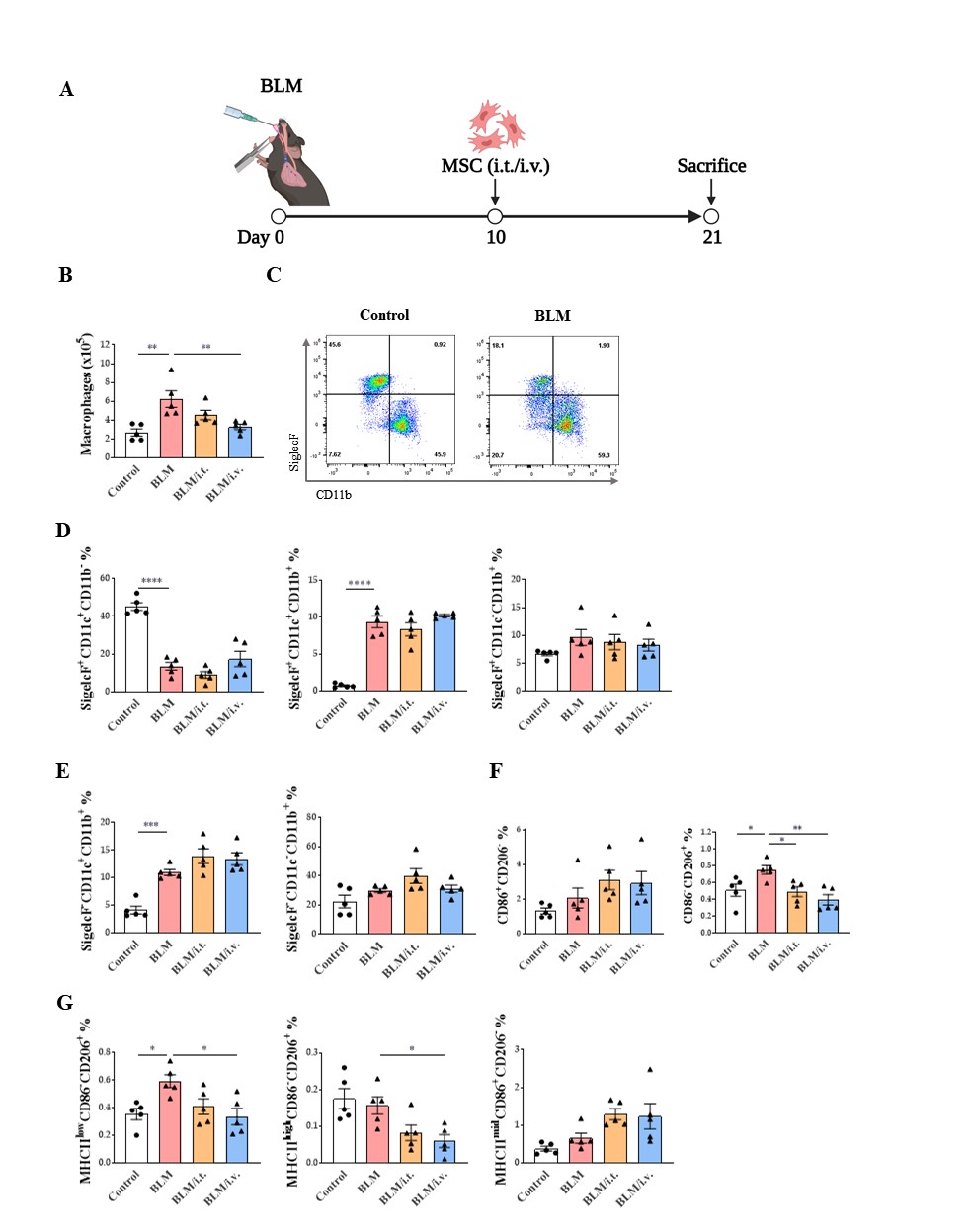


# Fig. S7. Effect of MSCs on the activation of subtypes of DCs in a murine fibrosis model

(A) Dot plot showing lung CD11b^+^ DCs population *in vivo*. (B) Changes in activation of CD11b^+^ DCs and cDC2s. n=5 for each group, * indicates *P* < 0.05, ** indicates *P* < 0.01, *** indicates *P* < 0.001, **** indicates *P* < 0.0001. All results are representative of at least three independent experiments. DC, dendritic cell; cDC2, type 2 conventional DCs


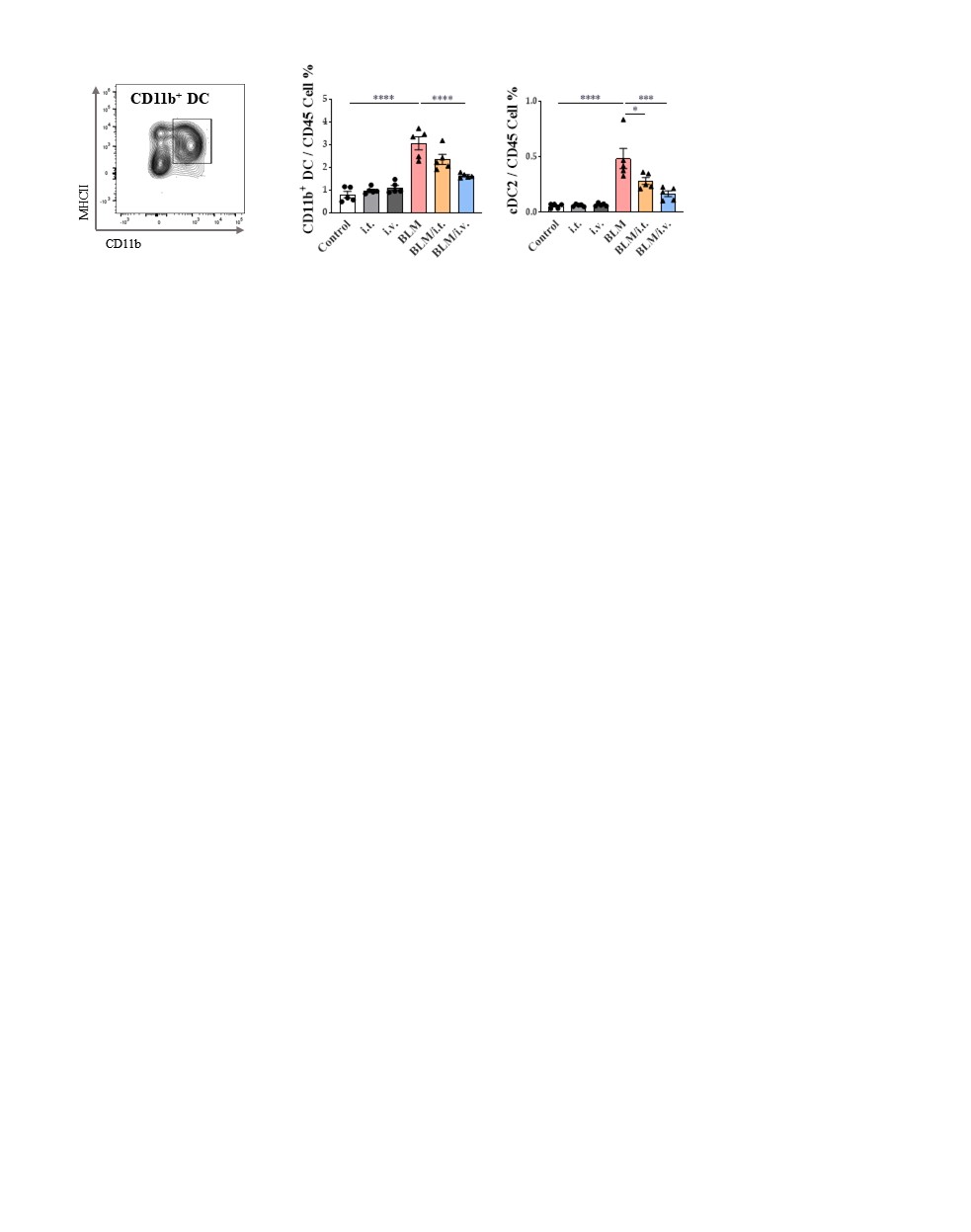


# Fig. S8. Regulatory effect of MSC on activation of control-derived Ly6c^+^ or Ly6c^-^ macrophages *ex vivo*

(A) Macrophage differentiation protocol of Ly6c^+^ or Ly6c^-^ monocytes isolated from control. (B) mRNA levels of macrophage activation markers, fibrosis-related markers, and immune cell chemotaxis markers in Ly6c^+^ or Ly6c^-^ MoM. n =4 for each group, * indicates *P* < 0.05, ** indicates *P* < 0.01, *** indicates *P* < 0.001, **** indicates *P* < 0.0001. All results are representative of at least three independent experiments. MSC, mesenchymal stem cell; MoM, monocyte-derived macrophage


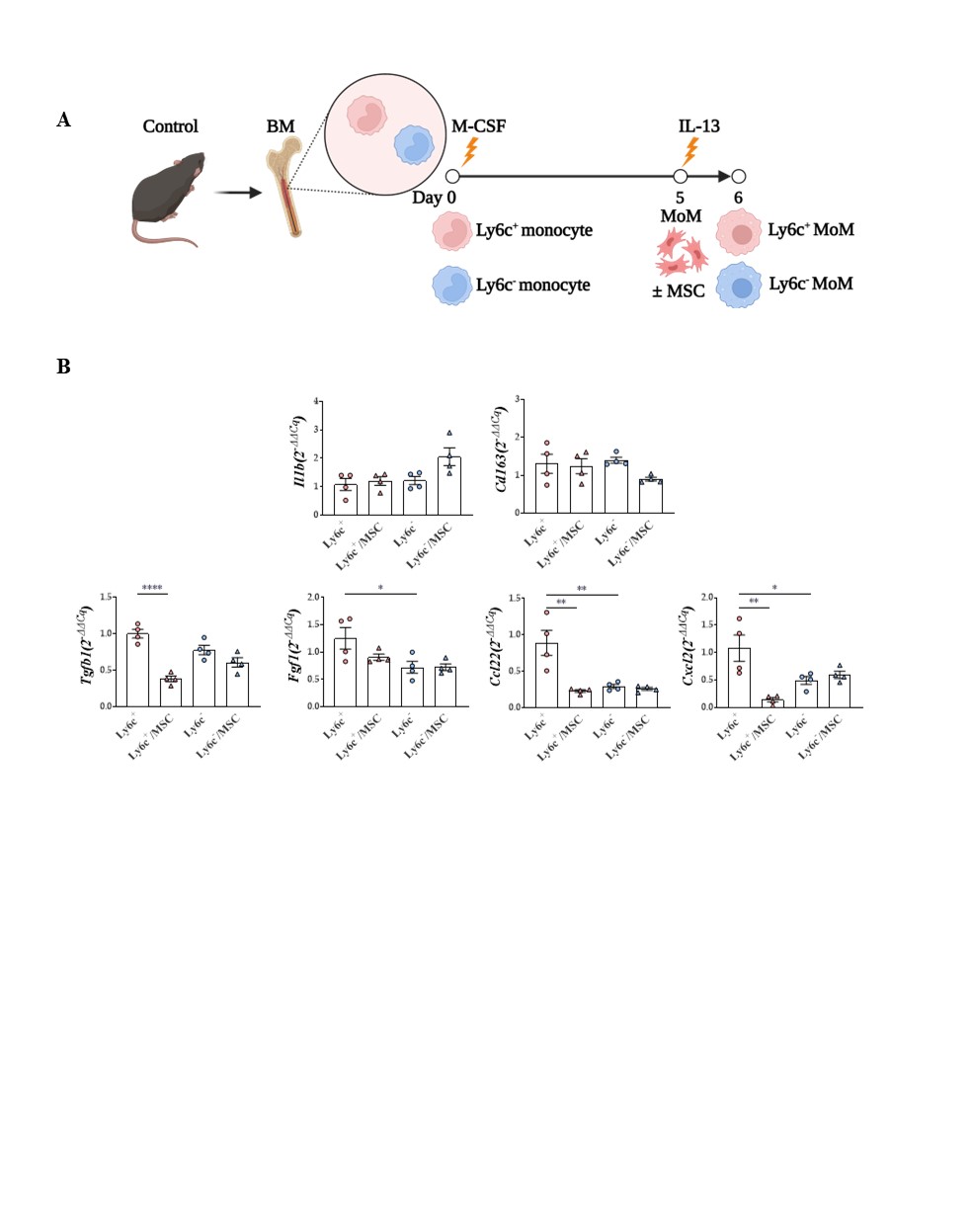


**Additional file 1: Table**

Table S1. Flow cytometry antibody list

| **FcγR block staning** | |
| --- | --- |
| **Name** | **Company** |
| Purified Rat Anti-Mouse CD16/CD32 | BD Biosciences |
| Human BD Fc Block^TM^ | BD Biosciences |

|  | **Macrophage staining** |  |
| --- | --- | --- |
| **Name** | **Fluorescence** | **Company** |
| MHCⅡ(Ⅰ-Ad) | APC | eBioscience  (eBioscienced, San Diego, CA) |
| CD45 | BV650 |  |
| SiglecF | BV421 |  |
| Ly6c | BV510 |  |
| CD11c | BV711 | Biolegend |
| CD11b | BV785 | (Biolegend, San Diego, CA) |
| CD86 | FITC |  |
| F4/80 | PE |  |
| CD206 | PE-Cy7 |  |
| MHCⅡ(Ⅰ-Ad) | APC | eBioscience |
| CD45 | BV650 |  |
| CD11c | BV421 |  |
| CD11b | BV785 | Biolegend |
| CD68 | FITC |  |
| CD206 | CD15 |  |

| **CD8^+^ T cell & T regulatory cell staining** | | |  |
| --- | --- | --- | --- |
| **Name** | **Fluorescence** | **Company** |  |
| CD45 | BV650 |  |  |
| IFN-γ | APC |  |  |
| CD25 | BV421 |  |  |
| CD4 | BV785 | Biolegend |  |
| CD3 | FITC |  |  |
| CD8 | PE |  |  |
| Foxp3 | PerCP-Cy5.5 |  |  |

|  | **Monocyte staning** |  |
| --- | --- | --- |
| **Name** | **Fluorescence** | **Company** |
| CD45 | BV650 |  |
| CD11b | APC |  |
| CD14 | FITC | Biolegend |
| CD16 | PE |  |
| CD15 | PerCP-Cy5.5 |  |

|  | **CD4^+^ T cell & ILC staning** |  |
| --- | --- | --- |
| **Name** | **Fluorescence** | **Company** |
| CD45 | BV650 |  |
| IL-5 | APC |  |
| IL-17 | BV421 |  |
| IFN-γ | PerCP-Cy5.5 | Biolegend |
| CD4 | BV785 |  |
| Lineage | FITC |  |
| IL-13 | PE |  |
| CD90.2 | PE-Cy7 |  |

Table S2. Primer sequences used in qPCR amplification

| **Gene** | **Human primer sequence** |
| --- | --- |
| *Gapdh* | Forward primer: 5’-AGACTCCACGACATACTCAG-3’  Reverse primer: 5’-ACGGCAAATTCAACGGCACA-3’ |
| *Hprt* | Forward primer: 5’-CTGGTGAAAAGGACCTCTCGAAG-3’ Reverse primer: 5’-CCAGTTTCACTAATGACACAAACG-3’ |
| *IL13* | Forward primer: 5’-GATCTGTGTCTCTCCCTCTGA-3’  Reverse primer: 5’-GTCCACACTCCATACCATGC-3’ |
| *Fn1* | Forward primer: 5’-TACCAAGGTCAATCCACACCCC-3’  Reverse primer: 5’-CAGATGGCAAAAGAAAGCAGAGG-3’ |
| *Gata3* | Forward primer: 5’-CCAGCTCACAGTATGGG-3’  Reverse primer: 5’-CCAGCTCACAGTATGGG-3’ |
| *Rorc* | Forward primer: 5’-CCGCTGAGAGGGCTTCAC-3’  Reverse primer: 5’-TGCAGGAGTAGGCCACATTACA-3’ |
| *Fgf1* | Forward primer: 5’-GGGAGATCACAACCTTCGCA-3’  Reverse primer: 5’-CTGTCCCTTGTCCCATCCAC-3’ |
| *Ccl11* | Forward primer: 5’-CCATGAAACCCACTGCCAT-3’  Reverse primer: 5’-GGAAGCTCTTTCTTCAAGGTG-3’ |
| *Il17a* | Forward primer: 5’-GAAGGCAGGAATCACAATC-3’  Reverse primer: 5’-GCCTCCCAGATCACAGA-3’ |
| *Cd86* | Forward primer: 5’-CAGACTCCTGTAGACGTGTTC-3’  Reverse primer: 5’-AACAGCATCTGAGATCAGCA-3’ |
| *Mrc1* | Forward primer: 5’-TATCTCTGTCATCCCTGTCTCT-3’  Reverse primer: 5’-CAAGTTGCCGTCTGAACTGA-3’ |
| *Mmp9* | Forward primer: 5’-CTTCTGGCGTGTGAGTTTCCA-3’  Reverse primer: 5’-ACTGCACGGTTGAAGCAAAGA-3’ |
| *Tbx-21* | Forward primer: 5’-AGCAAGGACGGCGAATGTT-3’  Reverse primer: 5’-GGGTGGACATATAAGCGGTTC-3’ |
| *Itgam* | Forward primer: 5’-ACGTCAGTACAAGGAGATGTTGGA-3’ Reverse primer: 5’-TCCCCATTCACGTCTCCCA-3’ |
| *IL10* | Forward primer: 5’-GGGTTGCCAAGCCTTATCG-3’  Reverse primer: 5’-TCTCACCCAGGGAATTCAAATG-3’ |
| *Il5* | Forward primer: 5’-GCCTCCCAGATCACAGA-3’  Reverse primer: 5’-CAGGAACAGGAATCCTCAGA-3’ |
| *Ym1* | Forward primer: 5’-AGAAGGGAGTTTCAAACCTGGT-3’  Reverse primer: 5’-GTCTTGCTCATGTGTGTAAGTGA-3’ |
| *Cxcl1* | Forward primer: 5’-CTGGCCACAGGGGCGCCTATC-3’  Reverse primer: 5’-GGACACCTTTTAGCATCTTT-3’ |
| *Cxcl2* | Forward primer: 5’-CCAACCACCAGGCTACAGG-3’  Reverse primer: 5’-GCGTCACACTCAAGCTCTG-3’ |
| *Ifng* | Forward primer: 5’-GGCCATCAGCAACAACATAAGCGT-3’ Reverse primer: 5’-TGGGTTGTTGACCTCAAACTTGGC-3’ |
| *Cd163* | Forward primer: 5’-ACTCTGAAGCGACGACAGATT-3’  Reverse primer: 5’-TGAATGACCCCCGAGGATTT-3’ |
| *Ccl22* | Forward primer: 5’-TACATCCGTCACCCTCTGCC-3’  Reverse primer: 5’-CGGTTATCAAAACAACGCCAG-3’ |
| *Foxp3* | Forward primer: 5’-CACCCAGGAAAGACAGCAACC-3’  Reverse primer: 5’-GCAAGAGCTCTTGTCCATTGA-3’ |
| *Tgfb* | Forward primer: 5’-AGGAGACGGAATACAGGGCT-3’  Reverse primer: 5’-CCACGTAGTAGACGATGGGC-3’ |
| *Col4a1* | Forward primer: 5’-GTCTGGCTTCTGCTGCTCTTC-3’  Reverse primer: 5’-CCTTCACGCCATGACAGTCA-3’ |
| *Il1b* | Forward primer: 5’-GCAACTGTTCCTGAACTCAACT-3’  Reverse primer: 5’-ATCTTTTGGGGTCCGTCAACT-3’ |
| *Fgf2* | Forward primer: 5’-GCGACCCACACGTCAAACTA-3’  Reverse primer: 5’-CCGTCCATCTTCCTTCATAGC-3’ |
| *Itgax* | Forward primer: 5’-ACGTCAGTACAAGGAGATGTTGGA-3’ Reverse primer: 5’-ATCCTATTGCAGAATGCTTCTTTACC-3’ |
| *HPRT* | Forward primer: 5’-TGGTCAGGCAGTATAATCCAAAGA-3’ Reverse primer: 5’-GTCAAGGGCATATCCTACAACAAAC-3’ |
| *HLA-DR* | Forward primer: 5’-TTTCCGCAAGTTCCACTATCTCCC-3’ Reverse primer: 5’-AATAATGATGCCCACCAGACCCAC-3’ |
| *RORC* | Forward primer: 5’-GCAGCGCTCCAACATCTTCT-3’  Reverse primer: 5’-ACGTACTGAATGGCCTCGGT-3’ |
| *CCR2* | Forward primer: 5’-ATGCTGTCCACATCTCGTTCTCG-3’  Reverse primer: 5’-TTATAAACCAGCCGAGACTTCCTG C-3’ |
| *FN1* | Forward primer: 5’-TCTGTGCCTCCTATCTATGTGC-3’  Reverse primer: 5’-GAGGGACCACGACAACTCTTC-3’ |
| *ACTA2* | Forward primer: 5’-GACAATGGCTCTGGGCTCTGTAA-3’ Reverse primer: 5’-ATGCCATGTTCTATCGGGTACTT-3’ |
| *TGFB1* | Forward primer: 5’-CCCAGCATCTGCAAAGCTC-3’  Reverse primer: 5’-GTCAATGTACAGCTGCCGCA-3’ |
| *CCL2* | Forward primer: 5’-GAGAGGCTGAGACTAACCCAGA-3’  Reverse primer: 5’-ATCACAGCTTCTTTGGGACACT-3’ |
| *CD163TGAX* | Forward primer: 5’-CAGCCTTTGACCTTATGTCATGG-3’  Reverse primer: 5’-CCTGTGCTGTAGTCGCACT-3’ |
| *ITGAX* | Forward primer: 5’-CGTTCGACACATCCGTGTA-3’  Reverse primer: 5’-TTTGCCTCCTCCATCATTTC-3’ |
| *ITGAM* | Forward primer: 5’-CAGCCTTTGACCTTATGTCATGG-3’  Reverse primer: 5’-CCTGTGCTGTAGTCGCACT-3’ |
| *MRC1* | Forward primer: 5’-GCAAAGTGGATTACGTGTCTTG-3’  Reverse primer: 5’-CTGTTATGTCGCTGGCAAATG-3’ |
| *IL13* | Forward primer: 5’-TGAGGAGCTGGTCAACATCA-3’  Reverse primer: 5’-CAGGTTGATGCTCCATACCAT-3’ |
| *IL5* | Forward primer: 5’-AGCTGCCTACGTGTATGCCA-3’  Reverse primer: 5’-GCAGTGCCAAGGTCTCTTTCA-3’ |
| *IL17* | Forward primer: 5’-GGACTGTGATGGTCAACCTGA-3’  Reverse primer: 5’-TCATGTGGTAGTCCACGTTCC-3’ |
| *CD86* | Forward primer: 5’-TGCTCATCTATACACGGTTAC-3’  Reverse primer: 5’-TTTCTTGGTCTGTTCACTCTC-3’ |
| *COL1A1* | Forward primer: 5’-ATGTCTAGGGTCTAGACATGTTCA-3’ Reverse primer: 5’-CCTTGCCGTTGTCGCAGACG-3’ |

**References**

1. Jeong EM, Shin JW, Lim J, Kim JH, Kang H, Yin Y, et al. Monitoring Glutathione Dynamics and Heterogeneity in Living Stem Cells. Int J Stem Cells. 2019;12(2):367-79.

2. Shin JW, Ryu S, Ham J, Jung K, Lee S, Chung DH, et al. Mesenchymal Stem Cells Suppress Severe Asthma by Directly Regulating Th2 Cells and Type 2 Innate Lymphoid Cells. Mol Cells. 2021;44(8):580-90.
